# PLK1 as a cooperating partner for BCL2-mediated antiapoptotic program in leukemia

**DOI:** 10.1038/s41408-023-00914-7

**Published:** 2023-09-07

**Authors:** Kinjal Shah, Ahmad Nasimian, Mehreen Ahmed, Lina Al Ashiri, Linn Denison, Wondossen Sime, Katerina Bendak, Iryna Kolosenko, Valentina Siino, Fredrik Levander, Caroline Palm-Apergi, Ramin Massoumi, Richard B. Lock, Julhash U. Kazi

**Affiliations:** 1 Division of Translational Cancer Research, Department of Laboratory Medicine, Lund University, Lund, Sweden; 2 Lund Stem Cell Center, Department of Laboratory Medicine, Lund University, Lund, Sweden; 3 Children’s Cancer Institute, Lowy Cancer Research Centre, School of Clinical Medicine, UNSW Medicine & Health, Centre for Childhood Cancer Research, UNSW Sydney, Sydney, NSW Australia; 4 Department of Laboratory Medicine, Biomolecular & Cellular Medicine, Karolinska Institutet, Stockholm, Sweden; 5 Department of Immunotechnology, Lund University, Lund, Sweden; 6 National Bioinformatics Infrastructure Sweden (NBIS), Science for Life Laboratory, Lund University, Lund, Sweden

**Keywords:** Acute lymphocytic leukaemia, Drug development

## Abstract

The deregulation of BCL2 family proteins plays a crucial role in leukemia development. Therefore, pharmacological inhibition of this family of proteins is becoming a prevalent treatment method. However, due to the emergence of primary and acquired resistance, efficacy is compromised in clinical or preclinical settings. We developed a drug sensitivity prediction model utilizing a deep tabular learning algorithm for the assessment of venetoclax sensitivity in T-cell acute lymphoblastic leukemia (T-ALL) patient samples. Through analysis of predicted venetoclax-sensitive and resistant samples, PLK1 was identified as a cooperating partner for the BCL2-mediated antiapoptotic program. This finding was substantiated by additional data obtained through phosphoproteomics and high-throughput kinase screening. Concurrent treatment using venetoclax with PLK1-specific inhibitors and PLK1 knockdown demonstrated a greater therapeutic effect on T-ALL cell lines, patient-derived xenografts, and engrafted mice compared with using each treatment separately. Mechanistically, the attenuation of PLK1 enhanced BCL2 inhibitor sensitivity through upregulation of BCL2L13 and PMAIP1 expression. Collectively, these findings underscore the dependency of T-ALL on PLK1 and postulate a plausible regulatory mechanism.

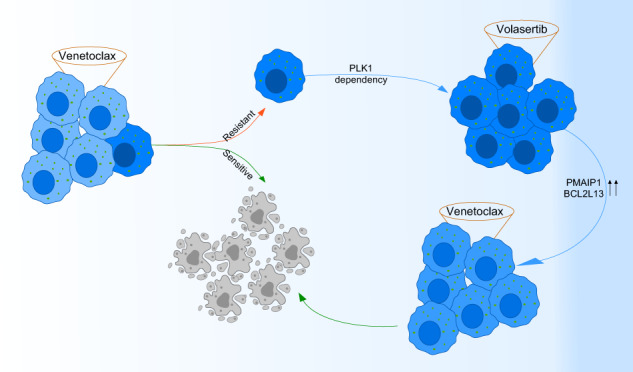

## Introduction

Despite its prevalent use in scientific research, machine learning exhibits considerable promise for predicting the efficacy of pharmacological responses with considerably high accuracy. This potential is achieved via the expeditious and precise identification of putative therapeutic targets and the development of patient-specific treatment approaches [1]. By implementing sophisticated computational algorithms, machine learning frameworks can proficiently analyze extensive quantities of multi-omics data, encompassing genomics, transcriptomics, proteomics, and metabolomics, to decipher complex patterns and associations [2]. These insights may be utilized to predict drug responses in individual patients, thereby fostering the advancement of precision medicine. Moreover, advanced deep learning approaches, including convolutional neural networks and recurrent neural networks, have considerably enhanced the predictive precision of pharmacological responsiveness models [3]. These cutting-edge methodologies can handle high-dimensional and complex data structures, effectively deciphering the nonlinear relationships between molecular features and pharmacological responses. Furthermore, machine learning models can determine the most relevant biomarkers and molecular pathways contributing to drug sensitivity by incorporating data integration and feature selection methods. This knowledge assists in designing novel targeted therapies and repurposing existing drugs for new indications.

Despite the significant advancements in predicting drug sensitivity using deep learning, several limitations and challenges persist. These challenges must be addressed to fully exploit potentials of deep learning algorithms in clinical applications [4, 5]. For example, data scarcity and heterogeneity represent major challenges of deep learning. High-quality, large-scale datasets are vital for training deep learning models; however, existing datasets often exhibit limited size, batch effects, and variability due to discrepancies in experimental conditions and platforms. Such heterogeneity may impede the application of models across diverse patient demographics and disease contexts.

Integrating multi-omics data, including genomics, transcriptomics, proteomics, and metabolomics, can augment drug sensitivity predictions [6]. Nevertheless, effectively assimilating these heterogeneous data sources remains challenging, necessitating the development of innovative algorithms and techniques to derive meaningful information and capture the complex relationships between data types. In addition, developing deep learning models capable of accounting for factors such as genetic background, tumor heterogeneity, and comorbidities and stratifying patients based on their predicted drug responses remains challenging [7]. Therefore, addressing these gaps through developing novel algorithms, generating high-quality datasets, and establishing standardized evaluation frameworks is crucial for advancing the application of deep learning in drug sensitivity prediction and promoting its integration into clinical practice. We applied TabNet as the core algorithm. TabNet is a deep learning model based on attention mechanisms, which has the potential to overcome several limitations associated with drug sensitivity prediction in clinical applications, including data scarcity, heterogeneity, and integration of multi-omics data [8]. It offers a potential solution for data scarcity and heterogeneity through its ability to learn meaningful representations even from small and noisy datasets. By leveraging attention mechanisms, the algorithm identifies and focuses on the most relevant features, mitigating the effects of batch variability and experimental discrepancies. This property enables TabNet to be applied across diverse patient demographics and disease contexts, making it suitable for clinical applications.

In this study, the model to predict venetoclax sensitivity was applied in T-cell acute lymphoblastic leukemia (T-ALL). T-ALL is one of the most aggressive forms of acute leukemia developing from immature lymphoid cells [9], and it accounts for ~15–25% of acute lymphoblastic leukemia (ALL) cases in children and adults, respectively [10]. In addition, it exhibits several distinctive genomic features that alter core signaling pathways, such as the deregulated expression of transcription factors, aberrant activation of notch signaling, loss of function mutations and deletions of tumor suppressors, activation of several kinase and cytokine signaling pathways, and cell cycle regulation [9]. Currently, the majority of T-ALL patients are treated with cytotoxic chemotherapy [11, 12]. Although the overall survival rate has increased in children due to improved risk assessment and chemotherapy combinations, T-ALL is highly refractory to chemotherapy upon relapse and has minimal therapeutic options [13]. Therefore, new treatments are required for this group of patients.

Residue cancer cells often act as a reservoir for refractory diseases [14]. They comprise a small fraction of cancer cells that resist programmed cell death (apoptosis) during drug treatment. The mechanisms of intrinsic and extrinsic apoptotic pathways have been studied in detail in the past decades. The BCL2 protein family tightly regulates the intrinsic apoptosis pathway by balancing the proapoptotic and antiapoptotic members [15]. Proapoptotic members act as sequesters (BAD, NOXA, etc.) of antiapoptotic members (BCL2, BCL-XL, MCL1, etc.) or as activators (BID, BIM, and PUMA) of mitochondrial pore-forming members (BAX, BAK, and BOK). Therefore, they facilitate mitochondrial outer membrane permeabilization (MOMP) and cytochrome C release. The upregulation of antiapoptotic members, such as BCL2, occurs in various cancers, including ALL, leading to resistance to therapy-induced apoptosis. Therefore, the inhibition of BCL2 has been considered to be a viable approach to eradicating residual cancer cells.

During the normal development of T-cells, BCL2 expression is biphasic; BLC2 expression is high in double-negative hematopoietic stem cells, low in most double-positive thymocytes, and high again in mature single-positive thymocytes [16, 17]. Similarly, BCL2 expression varies in T-ALL, with early T-cell progenitor ALL (ETP-ALL) displaying a higher level of BCL2 expression than non-ETP T-ALL [18]. Therefore, the ETP-ALL subtype shows a considerably higher response to BCL2 inhibition [19]. Numerous studies have shown that sensitivity to the BCL2-specific inhibitor venetoclax is dependent on a high BCL2 expression level [20–23]. However, the BCL2 expression levels do not always enable the prediction of treatment outcomes. For example, cells expressing lower levels of BCL2 also display venetoclax sensitivity [22, 23], while cells expressing a higher level of BCL2 can maintain significant venetoclax resistance [18–24]. Apart from the BCL2 expression level, it has been demonstrated that the expression of BCL-XL or MCL1 can determine BCL2 inhibitor sensitivity. Cells expressing higher BCL-XL or MCL1 show resistance to venetoclax; thus, an inhibitor targeting BCL2 and BCL-XL or MCL1 displays better efficacy in those cells [18, 25, 26]. However, this type of inhibition seems to have strong side effects [27, 28]. Therefore, besides those noteworthy developments, a better understanding of BCL2 inhibitor resistance is required to develop effective, safer treatments.

The present study devised an advanced tabular deep learning technique to autonomously fine-tune model parameters. We applied the model to predict venetoclax sensitivity, and by combining venetoclax sensitivity data with phosphoproteomics and high throughput drug screening, we ascertained that Polo-like kinase 1 (PLK1) assists in conferring venetoclax resistance.

## Methods

### Apoptosis assay

MOLT-16, LOUCY, and ALL-SIL cells were treated with DMSO and the EC_50_ (nM) of venetoclax, volasertib, and a combination in 6-well plates for 24, 48, and 72 h. Then, JURKAT, DND-41, CML-T1, TALL-1, and RPMI-8402 were treated in 6-well plates for 72 h with DMSO, venetoclax (50, 100, and 500 nM), and the respective concentrations of volasertib for each cell line along with the combination treatment. After each incubation period, the cells were processed, and the apoptotic cells were quantified using the FITC-Annexin-V/7-AAD kit (BD Biosciences, USA) according to the manufacturer’s protocol. Finally, the data were analyzed with the FlowJo software.

### Cell cycle analysis

MOLT-16, LOUCY, and ALL-SIL cells were treated with DMSO and the EC_50_ (nM) of venetoclax, volasertib, and combination in 6-well plates for 24, 48, and 72 h. Following incubation, cells were fixed with pre-chilled 70% ethanol, and the samples were stored at −20 °C. Then, the cells were treated with 100 µg/mL RNaseA and stained 50 µg/mL propidium iodide. After that, a flow cytometer was used to acquire signals from different cell populations. Finally, the cell cycle data were analyzed using the FlowJo software.

### Mouse xenograft studies

Five to seven-week-old nonobese diabetic or severe combined immunodeficient γ (NSG) mice (housed by the Laboratory Animal Facilities at Medicon Village, Lund University) were injected with 100 μL phosphate-buffered saline containing 2.5 million iRFP-positive DND-41 cells (also expressing luciferase, pHIV-iRFP720-E2A-Luc plasmid [29] was used, which was obtained from Addgene) through the tail vein. Bioluminescence imaging was used to follow the engraftment of cells. Two weeks after injection, mice were randomly divided into four groups (DMSO control, venetoclax, volasertib, and combination) based on the intensity of the bioluminescent signal, and the treatments were initiated. Both venetoclax and volasertib were formulated in the following order: 5% DMSO, 40% polyethylene glycol 300, 5% Tween-80, and 50% saline. The mice received 20 mg venetoclax per kg body weight and 5 mg volasertib per kg body weight via intraperitoneal injection every other day. The treatment for all four groups continued until the mice reached their endpoint (ruffled fur, lethargy, improper gait) or reached 20 drug injections. The mice were euthanized when they reached the endpoint. Furthermore, mice that survived 1 week after the last drug injection were considered alive when used in survival curves. All the animal experiments were performed under an ethical permit from the Swedish Animal Welfare Authority, and the mice were maintained following regulations approved by Lund University.

### Gene set enrichment analysis (GSEA)

Gene expression data of venetoclax-resistant and venetoclax-sensitive T-ALL cells were used to run a GSEA using the GSEA software (Broad Institute, USA). The Molecular Signatures Database was used to identify pathways enriched in predicted venetoclax-sensitive against venetoclax-resistant samples within those datasets. Moreover, GSEA was also used to identify pathways enriched in patient-derived xenografts (PDXs) that showed good or no synergy with venetoclax and volasertib.

### Statistical analysis

Statistical analysis was performed using the GraphPad Prism 5.0 (La Jolla, CA, USA) software, where data were expressed as mean ± SE. The unpaired Student’s *t* test and one-way ANOVA with Bonferroni’s post-test were used where applicable. Significance was set at *p* ≤ 0.05.

### PDX models and ex-vivo studies

The T-ALL PDX models have been described elsewhere [30]. Briefly, PDX cells were injected into immunodeficient NOD SCID gamma (NSG) mice via tail vein injection. Engraftments were assessed by blood sampling; mice were sacrificed when the proportion of CD45+ cells reached over 50%. Cells were collected from the spleen, bone marrow, and blood samples. For ex-vivo drug treatment, cells were seeded in 96-well plates using Stemline II Hematopoietic Stem Cell Expansion Medium (Sigma–Aldrich) supplemented with 10 ng/mL FLT3 ligand. Then, PDX cells were treated with different concentrations of venetoclax, volasertib, and combination for 48 h. Cell viability was measured using CellTiter-Glo (Promega) following the manufacturer’s instructions.

### Deep learning model and data

The binary classification models were developed as described previously [31–33], and pharmacogenomic data for venetoclax were retrieved from public databases. The IC_50_ value for venetoclax was used to define sensitivity, while cells with IC_50_values < 500 nM were classified as sensitive. Pharmacogenomic data were collected from BeatAML [34], scDEAL [35], and two more studies [36, 37].

### Additional methods

In supplementary methods.

## Results

### An autonomously fine-tuned deep tabular data learning model to predict drug sensitivity

In order to establish the drug sensitivity prediction model, we employed genes with significant variability derived from T-ALL patients’ data from the Therapeutically Applicable Research to Generate Effective Treatments (TARGET) database as the chosen attributes. Pharmacogenomic information, called annotated data, was acquired from studies such as scDEAL [35], BeatAML [34], GSE123883 [38], and GSE148715 [39]. In addition, non-annotated data (data lacking drug response information) were sourced from the BeatAML, The Cancer Genome Atlas Program, and TARGET repositories. Both annotated and non-annotated data with chosen attributes underwent a process of min-max normalization. Furthermore, the normalized annotated data were input directly into the sampling module for cross-validation assessment or segregated into test and training data subsets. Subsequently, the training data subset was transmitted to the sampling module.

**Fig. 1:**
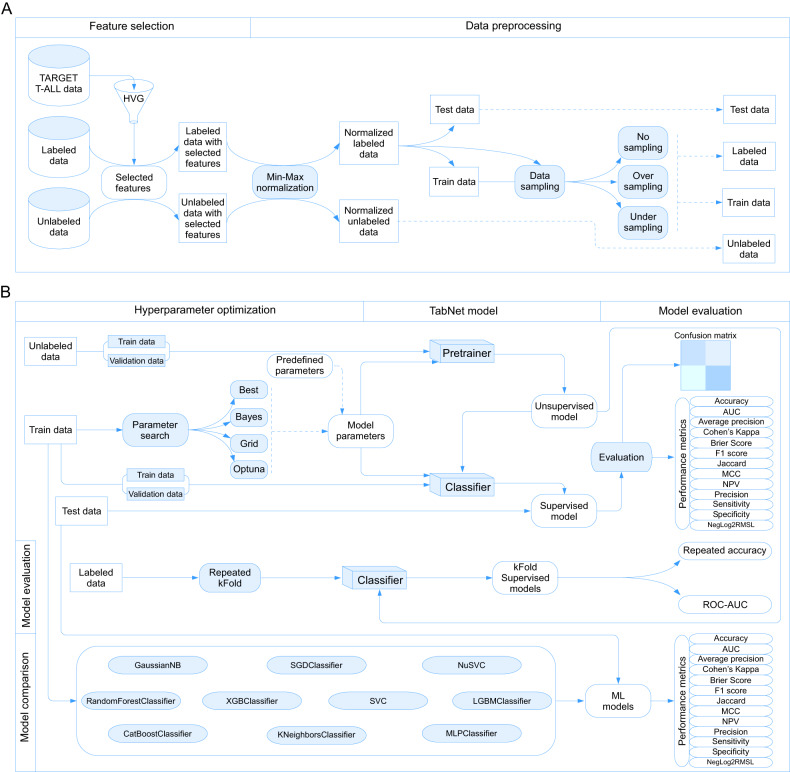
Comprehensive drug sensitivity prediction model development and evaluation pipeline using annotated and non-annotated data. **A** Workflow of the drug sensitivity prediction model development using annotated and non-annotated data. Annotated data were acquired from pharmacogenomic studies, while non-annotated data were sourced from various repositories. Both datasets underwent min-max normalization, and tripartite sampling strategies were applied to the training data subset. Test samples remained unaltered to prevent artificial influence on predictive outcomes. **B** Model training and evaluation pipeline incorporating hyperparameter optimization, TabNet pretraining, supervised TabNet model development, and performance assessment using multiple evaluation metrics. The pipeline also allows for comparative analysis with other machine learning models, offering adaptability for drug sensitivity prediction across diverse drug and cancer types.

A tripartite sampling strategy was implemented, encompassing unaltered samples, over sampling via the imbalanced-learn library, and under sampling through the same library. In every instance, test samples remained unaltered, ensuring that artificially generated data did not influence the predictive outcomes (Fig. 1A). Hyperparameter optimization was exclusively applied to the training dataset using three distinct methodologies: BayesSearchCV, GridSearchCV, and Optuna (Supplementary methods). Additionally, a pre-defined approach incorporating hardcoded hyperparameters was integrated, informed by previous experience or recommendations. A superior search option that utilized all four techniques was also implemented, yielding the optimal method based on a metric termed Negative Log_2_ Root Mean Square Loss (NegLog2RMSL). Finally, scoring was performed using combined Cohen’s Kappa-Matthews Correlation Coefficient (Cohen-MCC) metric.

Upon the optimization of hyperparameters, the TabNet pretraining algorithm was initially employed on unannotated data to construct an unsupervised model (Fig. 1B). Subsequently, the supervised TabNet model was developed by combining training samples with the weights obtained from the unsupervised model. After the establishment of the model, various evaluation metrics were employed for assessment. We employed a comprehensive suite of evaluation metrics, including Accuracy, Area Under the Curve (AUC), Average Precision, Cohen’s Kappa, Brier Score, F1 Score, Jaccard, MCC, Negative Predictive Value (NPV), Precision, Sensitivity, Specificity and NegLog2RMSL for testing the model. This multi-metric approach provides a more holistic evaluation of the model’s performance, given that different metrics address different aspects of a model’s predictive ability. By incorporating all these metrics, we can ensure a more robust and comprehensive assessment of our model, encompassing all critical aspects of its performance. Moreover, the option to generate repeated k-fold cross-validation models and evaluate them using the Receiver Operating Characteristic-Area Under the Curve and accuracy distribution was provided. For comparative analysis of model performance, an array of widely utilized machine learning models was integrated. The entire pipeline can be initiated via a single command line within a Jupyter Notebook environment, and by modifying the input data file, the model can be adapted for drug sensitivity prediction across diverse drug and cancer types.

### TabNet model predicting venetoclax sensitivity in T-ALL

**Fig. 2:**
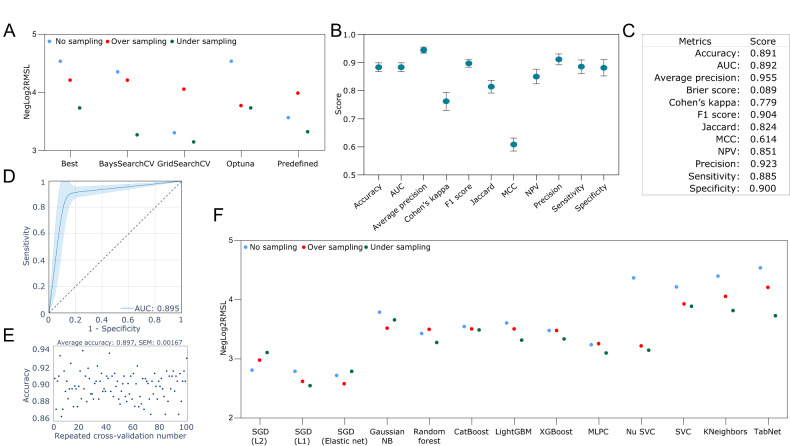
Performance comparison of TabNet models with different sampling and hyperparameter search methods. **A** NegLog2RMSL score comparison for different TabNet models. **B** Performance comparison among three sampling and hyperparameter search methods for TabNet models. **C** Summary of performance metrics for the TabNet model. **D** Average AUC was obtained from five-fold cross-validation with 20 repeated measurements. **E** Average accuracy was obtained from five-fold cross-validation with 20 repeated measurements. **F** Performance comparison of TabNet with other machine learning models.

Initially, a comparative analysis of TabNet models employing distinct sampling techniques and hyperparameter optimization strategies was conducted. Although there were minor discrepancies in the overall performance of some models as gauged by the NegLog2RMSL score (Fig. 2A), the variations in performance between the three sampling methods and hyperparameter optimization approaches were negligible (Fig. 2B and Supplementary Fig. 1A, B). The model achieved over 80% accuracy, AUC, average precision, F1 score, Jaccard index, NPV, precision, sensitivity, and specificity (Fig. 2C). The model’s predictions failed to identify 22 resistant cases (out of 191 samples) and 14 sensitive cases (out of 140 samples) (Supplementary Fig. 1C). Employing a five-fold cross-validation process with 20 repeated measurements yielded an average AUC of 0.895 (Fig. 2D) and an average accuracy of 0.897 (Fig. 2E), indicating the model’s resilience when applied to various sample subsets. TabNet model displayed comparable predictive performance to several other machine learning models (Fig. 2F).

### Venetoclax-resistant T-ALL displays enrichment in the PLK1 pathway

**Fig. 3:**
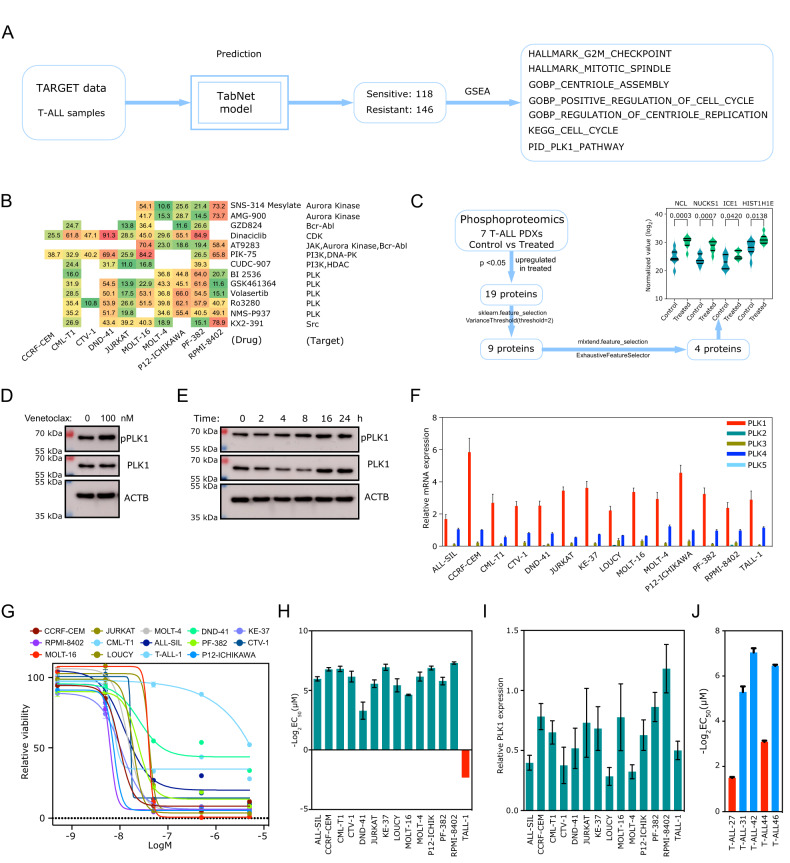
PLK1 dependency in venetoclax-resistant T-ALL. **A** Predicted venetoclax-sensitive and venetoclax-resistant patient samples were used to determine the gene set enrichment in the TARGET T-ALL cohort. **B** T-ALL cells were treated with the EC_33_ of BCL2 inhibitor and 100 nM of 378 kinase inhibitors. The numbers show that the percentage viability was reduced compared to the DMSO control (10% cut-off was used). **C** Seven T-ALL PDX-derived cell cultures were treated with BCL2 inhibitor or vehicle ex-vivo. Cells were lysed, and lysates were used for quantitative phosphoproteomics. Values were compared between treated and control samples (vehicle), and significantly upregulated phosphopeptides (*p* < 0.05) were selected as representative proteins. Then, the selected proteins were analyzed by sklearn.feature_selection (VarianceThreshold) following mlxtend.feature_selection (ExhaustiveFeatureSelector). **D**, **E** DND-41 cells were treated with venetoclax, and lysates were subjected to western blotting analysis. **F** Expression of PLK1-5 was determined using qPCR. **G**, **H** T-ALL cell lines were treated with different concentrations of volasertib for 48 h. Cell viability was measured using the PrestoBlue cell viability assay. GraphPad Prism was used to produce sigmoid curves and to determine EC_50_. **I** Protein expression of PLK1 was determined by western blots using specific antibodies. Band intensities were quantified using ImageJ. **J** T-ALL PDX cells were treated with different concentrations of volasertib for 48 h. Cell viability was measured and used to determine EC_50_ values.

The sensitivity of T-ALL to venetoclax was determined by employing the predictive model. Utilizing the TARGET dataset, comprising 264 samples, the model classified 118 samples as venetoclax-sensitive and 146 samples as venetoclax-resistant. After that, predicted samples were analyzed for pathway enrichment using Gene Set Enrichment Analysis (GSEA), and significant enrichment of cell cycle-associated pathways was detected, including the PLK1 pathway (Fig. 3A and Supplementary Tables 1–4).

Moreover, from an extensive panel of 378 kinase inhibitors, it was discovered that multiple PLK1-targeting inhibitors effectively potentiated the negative regulation of BCL2 inhibitor-induced cell viability (Fig. 3B). Additionally, a quantitative phosphoproteomic comparison of T-ALL PDXs treated with a BCL2 inhibitor or vehicle control revealed several proteins (Fig. 3C), such as Nucleolin (NCL) and Intraflagellar Transport 81 (IFT81, also known as ICE1), which have been reported as PLK1 substrates [40–43]. Moreover, the upregulation of PLK1 phosphorylation in venetoclax-treated cells was observed (Fig. 3D, E), suggesting that PLK1 may participate in the modulation of venetoclax sensitivity and targeted inhibition of PLK1 could potentially augment BCL2 inhibitor efficacy.

The PLK family is composed of five members: PLK1, PLK2, and PLK3, which share structural similarities, while PLK4 and PLK5 are considered distantly related [44]. Consequently, PLK1-specific inhibitors impede PLK2 and PLK3 function but exhibit limited efficacy in inhibiting PLK4 and PLK5 [45]. T-ALL cells predominantly express PLK1, whereas the expression of other PLK family members appears to be relatively low, except for some cells displaying notably elevated PLK4 expression (Fig. 3F). Most T-ALL cell lines assessed demonstrated heightened sensitivity (EC50 below 100 nM) to the PLK1 inhibitor volasertib, except for TALL-1, which exhibited a poor response (Fig. 3G, H). PLK1 protein expression varied among the cell lines (Fig. 3I) and exhibited a weak correlation with PLK1 inhibitor sensitivity. Additionally, T-ALL PDXs displayed a heterogeneous response to the inhibitor, with certain PDXs being highly sensitive (EC_50_: approximately 25 nM), while others were less sensitive (EC_50_: ~120 and 350 nM) (Fig. 3J). The findings indicate that venetoclax-resistant T-ALL demonstrates PLK1 pathway enrichment and sensitivity to PLK1 inhibition, while venetoclax treatment promotes PLK1 phosphorylation.

### Venetoclax displays synergy with PLK1 inhibitor in T-ALL

**Fig. 4:**
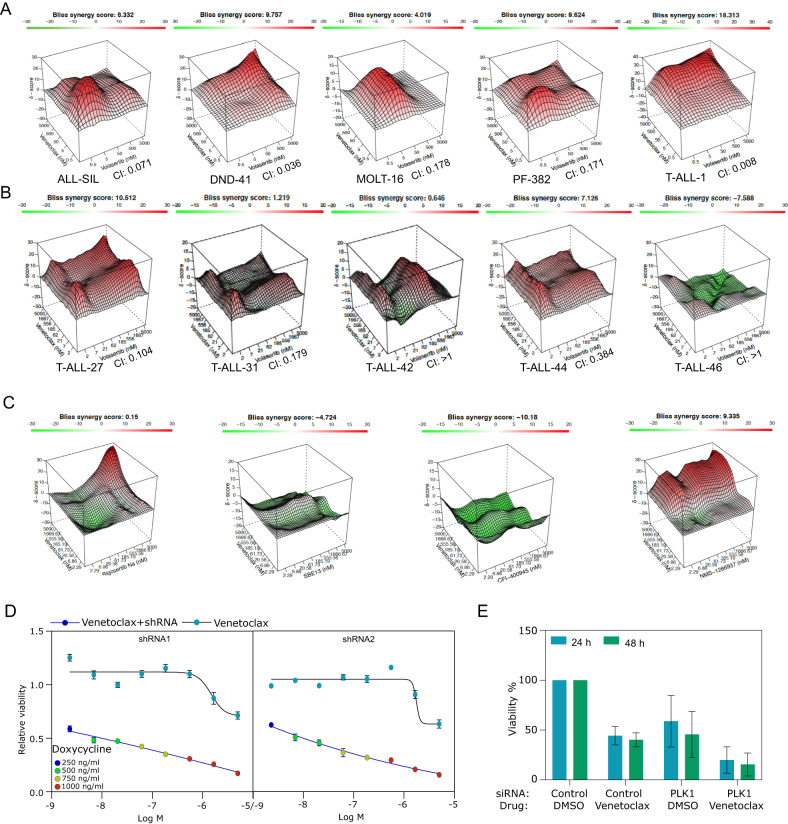
The synergy between venetoclax and volasertib. **A**, **B** T-ALL cell lines or PDX cells were treated with different concentrations of venetoclax, volasertib, or 1:1 combination for 48 h to determine synergy scores. **C** The synergy between venetoclax and other PKL-related inhibitors in DND-41 cells was measured similarly. **D** DND-41 cells were stability transfected with inducible PLK1 shRNA. Cells were then treated with doxycycline to induce PLK1 shRNA expression. Venetoclax was added to observe the synergistic effect. **E** DND41 cells were transfected with 100 nM siRNA. 4 h after transfection, the media was changed to regular growth media and supplemented with vehicle or Venetoclax (5 μM). Aliquots were taken 24 h and 48 h after transfection to assess cell viability by CellTiterGlo.

Owing to the observed differential response of T-ALL cell lines and PDXs to volasertib, this study sought to investigate the potential of venetoclax to augment the effects of the PLK1 inhibitor. Previous research demonstrated synergism between PLK1 and BCL2 inhibitors in double-hit lymphoma [46]. Therefore, the synergistic effects were assessed by employing a broad spectrum of concentrations, and it was observed that T-ALL cell lines exhibited synergy upon combining venetoclax with volasertib (Fig. 4A and Supplementary Fig. 2A). Analogous to the cell lines, the PDXs T-ALL-27 and T-ALL-44 (exhibiting low sensitivity to the PLK1 inhibitor) displayed increased synergy as determined by both the BLISS score and combination index (Fig. 4B). In contrast, T-ALL-42 and T-ALL-46, which displayed high sensitivity to PLK1 inhibitors, demonstrated no synergistic effect. In addition, the non-specific PLK1 inhibitor rigosertib and SBE13, as well as the PLK4 inhibitor CFI-400945, exhibited no synergy, while the specific PLK1 inhibitor NMS-1286937 (Onvansertib) demonstrated synergy (Fig. 4C and Supplementary Fig. 2B). Moreover, the knockdown of PLK1 using inducible short hairpin RNAs (Supplementary Fig. 3A) or small interfering RNA (Supplementary Fig. 3B) displayed synergy with venetoclax (Fig. 4D, E), suggesting that PLK1 inhibition is essential for the synergistic interaction between venetoclax and volasertib.

### Combined use of venetoclax and PLK1 inhibitor enhances apoptosis and survival of mice injected with T-ALL cells

**Fig. 5:**
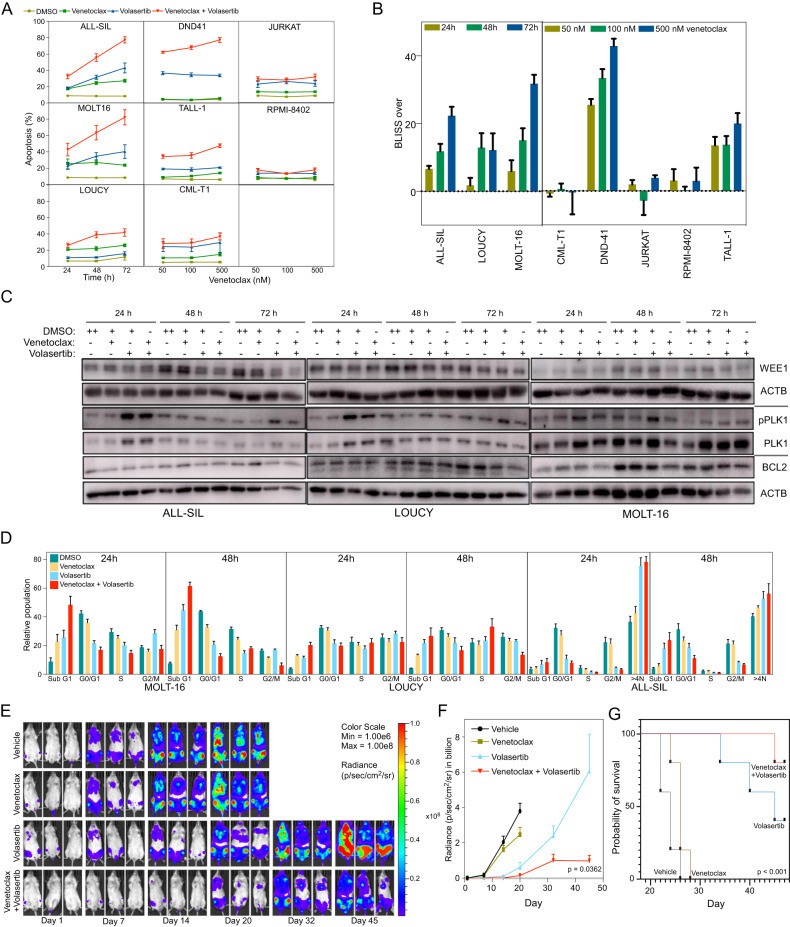
Venetoclax displays synergy with volasertib in apoptosis induction and increases the survival of T-ALL cells engrafted mice. **A** T-ALL cells were treated with venetoclax, volasertib, or a 1:1 combination at different intervals. Apoptosis was measured using an Annexin-V/7-AAD kit. **B** The Bliss Independence method was used to calculate the BLISS score with the following formula: observed combined effect − expected combined effect of the two drugs. The expected combined effect of the two drugs was calculated as follows: inhibitory effect of drug 1 (venetoclax) + inhibitory effect of drug 2 (volasertib) – inhibitory effect of drug 1 (venetoclax) * inhibitory effect of drug 2 (volasertib). **C** ALL-SIL cells were treated with the EC_50_ (nM) of venetoclax, volasertib, or a combination for different periods, after which they were lysed and probed with the indicated antibodies. **D** Cells were treated with DMSO and the EC_50_ (nM) of venetoclax, volasertib, or a combination for different time points. Then, the cells were stained with propidium iodide and analyzed by flow cytometry. **E**, **F** DND-41 cells carrying luciferase were injected into NSG mice through their tail vein. Luciferase activity was measured after 2 weeks (depicted as day 1 in the graph), and the mice were randomly selected for different treatments. **G** Kaplan-Meier curves showing the survival of the mice.

Upon observing the synergistic reduction of cell viability, we subsequently investigated whether the concurrent inhibition of PLK1 and administration of venetoclax could potentiate apoptosis. A synergistic enhancement of apoptosis was observed in ALL-SIL, DND-41, MOLT-16, and TALL-1 cells; however, this effect was not detected in the CML-T1, JURKAT, and RPMI-8402 cell lines (Fig. 5A, B). While BCL2 expression remained unaltered, PLK1 stabilization occurred in response to volasertib treatment (Fig. 5C). Cell cycle analyses revealed an increased Sub G1 population in cells treated with venetoclax and volasertib (Fig. 5D). Additionally, NSG mice engrafted with luciferase-expressing DND-41 cells exhibited reduced luciferase intensity and improved survival when venetoclax was administered with volasertib, compared with other treatment groups (Fig. 5E, F, G). These findings indicate that venetoclax synergizes with the PLK1 inhibitor in selected cells, particularly those with moderate sensitivity to PLK1 inhibition.

### PLK1 inhibition induces BCL2L13 and PMAIP1 expression

**Fig. 6:**
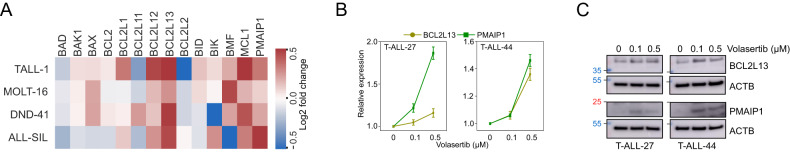
PLK1 inhibition enhances BCL2L13 and PMAIP1 expression. **A** Cells were treated with volasertib or DMSO before the collection of mRNA. qPCR was used to determine BCL2 family gene expression changes in response to volasertib. **B** PDXs were treated with different concentrations of volasertib for 24 h before the collection of mRNA. qPCR was used to determine the relative expression of the BCL2L13 and PMAIP1 genes. **C** Cells were treated with different concentrations of volasertib for 24 h before being lysed. Lysates were analyzed using SDS-PAGE.

Subsequently, this study investigated the potential underlying mechanism driving the synergistic interaction between venetoclax and volasertib. Initially, the impact of PLK1 inhibition on the transcriptional regulation of BCL2 family members was assessed. The expression levels of 21 BCL2 family genes were quantified utilizing RT-qPCR. Among these, seven genes exhibited low expression in T-ALL cells and were excluded from further analysis. Furthermore, the expression of several other BCL2 family genes remained unaltered following PLK1 inhibition. Notably, an upregulation in the expression of BCL2L13 (BCL-Rambo) and PMAIP1 (NOXA) was detected upon PLK1 inhibition (Fig. 6A). This finding was validated in T-ALL PDXs at the mRNA (Fig. 6B) and protein levels (Fig. 6C). BCL2L13, known to not cooperate with other family members, can induce apoptosis by interacting with mitochondrial permeability transition pore (MPTP) [47]. Conversely, PMAIP1 has been reported to augment venetoclax sensitivity [48, 49].

## Discussion

This study aimed to establish a drug sensitivity prediction model for T-ALL patients, using genes with significant variability derived from the TARGET database. For example, pharmacogenomic data for venetoclax showed that samples predicted by the model are useful for downstream analysis and identifying effective drug combinations.

A tripartite sampling strategy was implemented, and four hyperparameter optimization methods were applied to identify the best parameters for developing a deep tabular data learning model. Model performance was assessed using combined Cohen-MCC metric and NegLog2RMSL was used for optimizing the parameter for the best model. The TabNet algorithm was used as the core classification model for this study as it offers several advantages over conventional deep learning models [8]. First, TabNet automatically selects the most relevant features for the task. Then, it uses an attention mechanism to identify the most important variables and eliminate the need for manual feature engineering. The algorithm is designed to handle large datasets efficiently. Finally, unlike other deep learning algorithms, TabNet architecture is accessible for interpretation.

Model performance was assessed using various evaluation metrics and compared with other widely utilized machine learning models. The analysis revealed that although there were minor discrepancies in the overall performance of some models as gauged by the NegLog2RMSL score, the variations in performance between the three separate sampling methods and hyperparameter optimization approaches were negligible. Various performance metrics achieved a score of over 80%, including Accuracy, AUC, Average Precision, F1 score, Jaccard index, NPV, Precision, Sensitivity, and Specificity. Employing a five-fold cross-validation process with 20 repeated measurements demonstrated the model’s resilience when applied to various sample subsets. Comparatively, TabNet displayed comparable performances with the other machine learning models examined.

The sensitivity of T-ALL to venetoclax was determined using this study’s predictive model, and samples were classified as venetoclax-sensitive or venetoclax-resistant. Pathway enrichment analysis using GSEA revealed significant enrichment of cell cycle-associated pathways, including the PLK1 pathway. It was discovered that multiple PLK1-targeting inhibitors effectively enhanced the negative regulation of BCL2 inhibitor-induced cell viability. Furthermore, a quantitative phosphoproteomic comparison revealed several proteins, such as NCL and IFT81, reported as PLK1 substrates, which promote the upregulation of PLK1 phosphorylation in venetoclax-treated cells. This suggests that PLK1 may be involved in modulating venetoclax sensitivity and that targeted inhibition of PLK1 could potentially augment BCL2 inhibitor efficacy.

Intrinsic apoptosis pathways are tightly regulated by BCL2 family members. Antiapoptotic BCL2 members such as BCL2, BCL2L1, and MCL1 prevent apoptosis by sequestering proapoptotic BAK1, BAX, and BOK. When these proapoptotic components are released from their antiapoptotic partners, they facilitate MOMP. The deregulated expression of those antiapoptotic members is often associated with therapy resistance, and inhibiting them provides means for overcoming chemo- and immunotherapy resistance [50].

In clinical and preclinical settings, the highly specific BCL2 inhibitor venetoclax has been used for several hematological malignancies, including AML, CLL, B-ALL, and T-ALL. Due to the dynamic regulation of BCL2 expression during T-cell development, expression patterns vary in T-ALL; as a result, the response to venetoclax varies widely. As a result of high levels of BCL2 expression during the early development of T-cells, ETP-ALL displays a higher level of BCL2 and is highly responsive to venetoclax. In contrast, the response in non-ETP T-ALL remains inconclusive. This study demonstrates that venetoclax response can be regulated by pharmacological interference from PLK1 activity.

Even the most potent drug might be unable to remove a small portion of cancer cells, leading to minimal residual disease, which is the key player in acquired resistance. During drug therapy, these cells acquire genetic and non-genetic modifications that help them escape drug-induced apoptosis. Recent studies suggest that the cells driving drug resistance can be present even at the initial stages of the disease, and the genetic and non-genetic mechanisms of resistance development are not mutually exclusive. Likewise, resistance to venetoclax can emerge during treatment or be present at the beginning of treatment. Initially, it was suggested that venetoclax resistance is mediated by the differential expression of BCL2 and BCL2L1 proteins [18]. However, recent studies have demonstrated the involvement of several signaling proteins and pathways [51]. For example, AML resistance can be acquired through the upregulation of BCL2A1 and CLEC7A, mutations in PTPN11 and KRAS [52], upregulation of the mitochondrial chaperonin CLPB [53], and inactivation of TP53, BAX, and PMAIP1 genes [54]. Mechanistically, the inactivation of TP53 leads to the activation of the Ras/Raf/MEK/ERK pathway and, therefore, the inactivation of GSK3, preventing phosphorylation-dependent degradation of MCL1 in AML [55]. Several mechanisms, including AKT-mediated phosphorylation on serine nine, can inactivate GSK3. In aggressive B-cell lymphoma, venetoclax resistance is mediated by the abnormal activation of the PI3K/AKT pathway due to the compromised expression of PTEN; the inhibitors targeting this pathway display synthetic lethality [56]. This is probably also mediated by MCL1 stabilization through the inactivation of GSK3 by AKT. Several cellular regulatory processes, such as the modulation of lymphoid transcription and the AMPK/PKA axis, cellular energy metabolism, and overexpression of MCL1, contribute to venetoclax resistance in chronic lymphocytic leukemia [57]. MCL1 seems to be a key protein that mediates BCL2 inhibitor resistance, although several other proteins and pathways also contribute.

While studies suggest that ETP-ALL predominantly expresses high levels of BCL2, some non-ETP T-ALL patients also carry high levels of BCL2. Therefore, it is likely that this group of patients would show a clinical response to venetoclax. However, venetoclax response is not only determined by BCL2 expression. As discussed above, examples from other hematological malignancies suggest the involvement of several complicated cellular processes [52–57]. Therefore, improved biomarker-driven drug sensitivity prediction would be helpful in patient selection. This study developed a drug sensitivity prediction model using pharmacogenomic data for venetoclax and a deep learning model. The model allowed the prediction of venetoclax sensitivity using transcriptomic data that facilitated the identification of PLK1 as a possible mediator of venetoclax resistance. PLK1 is a well-studied serine-threonine kinase known for its function in mitosis and its role in cancer [58]. In T-ALL, PLK1 seems to be the dominant isoform, and an inhibitor targeting PLK1 displayed considerable inhibitory potential against cultured cell lines, ex-vivo treatments of PDXs, and a mouse xenograft model. The data are consistent with a previous study that discovered PLK1 expression and phosphorylation of its threonine 210 was considerably higher in T-ALL patients than in normal bone marrow mononuclear cells. In addition, shRNA mediated the knockdown of PLK1-induced apoptosis [59].

PLK1 mediates serine-threonine phosphorylation of the protein panel that regulates mitosis and modulates chromosome dynamics, apoptosis, and transcription [58]. PLK1 also holds the ability to modulate transcriptional programs positively and negatively. While PLK1-mediated phosphorylation of Forkhead Box M1 (FOXM1) regulates a transcriptional program required for G2/M transition [60], FOXO1- and FOXO3a-dependent transcription programs are repressed by PLK1-mediated phosphorylation [61, 62]. The transcription of several BCL2 family genes, including BCL2L11, PMAIP1, and BCL2L13, is regulated by FOXOs [47, 63, 64]. It was observed that PLK1 inhibition induces the expression of BCL2L13 and PMAIP1, suggesting that PLK1 mediates T-ALL cell survival by suppressing BCL2L13 and PMAIP1 expression, probably through FOXOs. PMAIP1 cooperates with BCL2 inhibition by sequestering BCL2L1 and MCL1, and BCL2L13 can directly induce mitochondrial apoptosis [47, 49]. The data in this study suggest that synergy between the BCL2 and PLK1 inhibitors is mediated by transcriptional regulation of BCL2–family proteins.

### Supplementary information

Supplementaal Materials

## Data Availability

For raw data, please send a request to the corresponding author.

## Code availability

Codes are available at https://github.com/kazilab/venDx.
