# Supplementary material for: PLK1 as a cooperating partner for BCL2-mediated antiapoptotic program in leukemia
Source: Blood Cancer J. 2023 Sep 7;13(1):139. doi: 10.1038/s41408-023-00914-7 (PMC10484999; doi:10.1038/s41408-023-00914-7)
Supplement: Supplementary file 1 — Supplementaal Materials [file 41408_2023_914_MOESM1_ESM.pdf]

PLK1 as a Cooperating Partner for BCL2-Mediated Antiapoptotic Program in Leukemia

Kinjal Shah, Ahmad Nasimian, Mehreen Ahmed, Lina Al Ashiri, Linn Denison, Wondossen Sime, Katerina Bendak, Iryna Kolosenko, Valentina Siino, Fredrik Levander, Caroline Palm-Apergi, Ramin Massoumi, Richard B. Lock and Julhash U. Kazi

Supplementary Materials

Supplementary Methods

Cell lines – The human T-ALL cell lines (CCRF-CEM, JURKAT, DND-41, MOLT-16, KE-37, CML-T1, PF-382, P12-ICHIKAWA, and RPMI-8402) were cultured in RPMI 1640 supplemented with 10% heat-inactivated fetal bovine serum (FBS) (Thermo Fisher Scientific, USA), 100 U/mL penicillin, and 100 µg/mL streptomycin (Corning, USA). In contrast, ALL-SIL, LOUCY, TALL-1, CTV-1, and MOLT-4 cell lines were cultured in RPMI 1640 supplemented with 20% heat-inactivated FBS (Thermo Fisher Scientific, USA), 100 U/mL penicillin, and 100 µg/mL streptomycin (Corning, USA). All cell lines were obtained from Deutsche Sammlung von Mikroorganismen und Zellkulturen (DSMZ, Braunschweig, Germany) and maintained in a Heraeus BBD 6220 Incubator (Thermo Fisher Scientific, USA) at 37°C with 5% CO2; the cells were routinely tested for mycoplasma.

Drug sensitivity assays – T-ALL cell lines were tested for their viability against different concentrations of the BCL2 inhibitors navitoclax (MedChemExpress USA) and venetoclax (MedChemExpress USA), and the PLK inhibitor volasertib (MedChemExpress USA), in 96-well plates, where cells were seeded at a density of 20,000 cells/well. Venetoclax-resistant MOLT-16, LOUCY, and ALL-SIL cells were seeded similarly to determine the effective concentration 50 (EC_{50}) (nM) for venetoclax. After 48-hour incubation, 10 µL PrestoBlue (Thermo Fisher Scientific, USA) was added to each well. Following a two-hour incubation, fluorescence was measured with a plate reader, and EC_{50} values were calculated using the GraphPad Prism software. A kinase inhibitor library of 378 kinase inhibitors was obtained from Selleck Chemicals (Houston, TX, USA). Also, a panel of 10 T-ALL cell lines (CCRF-CEM, CML-T1, CTV-1, DND-41, JURKAT, MOLT-16, MOLT-4, P12-ICHIKAWA, PF-382, and RPMI-8402) was seeded in 96-well plates containing 100 nM of each kinase inhibitor and EC_{33} (nM) of navitoclax for that particular cell line. The plates were read with PrestoBlue in the same way as for the cell viability assay. The percentage of growth inhibition was calculated as 100 minus the percentage of cell viability.

Western blot and antibodies – Cells were treated with DMSO, venetoclax, volasertib, or a combination for 24, 48, and 72 hours, followed by lysis in RIPA or Triton X-100 lysis buffer. Lysates were separated on SDS-PAGE gels following transfer to polyvinylidene difluoride (PVDF) membranes. The anti-WEE1 (sc-5285, 1:1000 dilution), anti-PLK1 (sc-17783, 1:500 dilution), anti-BCL2 (sc-509, 1:1000 dilution), and anti-β-actin-HRP (sc-47778, 1:2000 dilution) were obtained from Santa Cruz Biotechnology, USA. The anti-phospho-PLK1 (5472S, 1:1000 dilution) was obtained from Cell Signaling Technologies, USA. Anti-β-catenin (610153, 1:1000 dilution) came from BD Biosciences, USA. Anti-NOXA(PMAIP1, PRS2437-100UG, 1:1000 dilution) and Anti-BCL-rambo (BCL2L13, PRS3023-100UG, 1:1000) were obtained from Sigma-Aldrich, Germany. All the blots were derived from the same experiment and processed in parallel wherever possible. Lastly, the images were cropped for presentation.

Real-time quantitative PCR (RT-qPCR) – Total RNA was extracted from T-ALL cell lines in the presence or absence of volasertib treatment, using the RNeasy mini kit (Qiagen) following the manufacturer's instructions. RT-qPCR was used to assess gene expression, and each sample was analyzed in quadruplicate. Different probes used for RT-qPCR included PLK1, PLK2, PLK3, PLK4, PLK5, BCL2L13, PMAIP1 and three housekeeping genes. These probes were ordered from Thermo Fisher Scientific, USA. An array of probes consisting of 21 BCL2 family genes and three housekeeping genes were ordered from Thermo Fisher Scientific, USA.

Measuring of synergy between venetoclax and volasertib – T-ALL cell lines and PDX cells were treated with a range of venetoclax and volasertib concentrations and a 1:1 combination of both in 96-well or 384-well plates. After 48-hour incubation, cell viability was measured by either PrestoBlue (Thermo Fisher Scientific, USA) or the CellTiter-Glo Cell Viability Assay (Promega, Madison, WI, USA) following the manufacturer's protocol. A full synergy matrix was generated using the DECREASE web app [1]. The BLISS score was calculated by SynergyFinder [2], and the combination index (CI) was calculated using ComboSyn [3].

Phosphoproteomics – Approximately 150 µg protein extract per sample was subjected to overnight in-solution digestion using Lys-C and trypsin at an enzyme: protein ratio of 1:100 and 1:50, respectively. Then, 1% trifluoroacetic acid (TFA) was added to each sample, and the samples were dried before resuspension and desalting using C18-packed columns (Nest Group, Southborough, USA) according to the manufacturer's instructions. According to the

manufacturer's instructions, desalted peptides were phospho-enriched using Ti-IMAC (Resyn Bioscience) beads. After that, the Phosphopeptides were dried and resuspended in 0.1% FA prior to LC-MS/MS analysis with peptide separation on an EASY-nano LC 1200 system (Thermo Fisher Scientific, Germany) equipped with an in-house packed 15 cm long fused silica capillary (75 µm* 16 cm Pico Tip Emitter, New Objective) with C18 material ReproSil-Pur 1.9 µm (Dr. Maisch GmbH, Germany). Peptides were separated using a 50 min gradient from 5% to 90% solvent B (80% ACN, 0.1% FA) at a constant flow rate of 250 nl/min. The nanoLC system was coupled to a Q-Exactive HF-X Mass Spectrometer (Thermo Fisher Scientific, Germany) operated in positive ion mode for data-dependent acquisition (DDA). In addition, a top-20 method with fragmentation and MS/MS analysis of charge state 2 to 6 precursors was employed, with an MS1 target resolution of 120000 and MS2 target resolution of 15000. The raw data were converted to mzML and MGF with Proteowizard [4] and processed using the Proteios Software Environment [5]. MS/MS identification was performed using X!Tandem (www.thegpm.org) and MS-GF+ [6], feature detection was conducted using Dinosaur [7] with the workflow and settings mainly arranged as described previously [8]. Data were log2-transformed and quality-controlled using NormalyzerDE [9] before statistical analysis.

Min-max normalization – Min-max normalization is a widely employed data preprocessing technique that aims to standardize and scale numerical features within a specific range, typically 0 and 1. This transformation ensures that features with disparate magnitudes and units are comparable, enhancing the performance and convergence of various machine learning and statistical algorithms. The min-max normalization process involves linearly scaling each data point by applying the following formula:

normalized_value = (original_value – min_value) / (max_value – min_value)

In this case, "original_value" represents the data point in question, while "min_value" and "max_value" correspond to the minimum and maximum values within the feature, respectively. As a result of this transformation, the minimum value becomes 0, and the maximum value becomes 1, with all intermediate values proportionally scaled between these bounds.

Sampling method – imbalanced-lear, which is a Python library offering comprehensive tools and techniques, was used for addressing class imbalance issues in machine learning [10]. The library is built on top of sci-kit-learn, a prominent machine-learning library, and adheres to its conventions and API design [11]. Imbalanced-learn primarily provides algorithms for resampling imbalanced datasets through under-sampling or over-sampling. Under-sampling refers to reducing the number of instances in the majority class, effectively balancing the class distribution. This technique is beneficial when there is a significant difference in the number of samples between classes, as it prevents the model from being biased toward the majority class. Tomek links were incorporated with random under-sampling to achieve under-sampling.

Over-sampling, which involves increasing the number of instances in the minority class to create a more balanced dataset, was achieved using Synthetic Minority Over-sampling Technique (SMOTE). Additionally, a tertiary approach in which the sample population remained unaltered was incorporated, and class equilibrium was accomplished by conveying class weights directly to the model.

Hyperparameter search – Three methods for hyperparameter optimization was applied, including BayesSearchCV, GridSearchCV, and Optuna. BayesSearchCV employs Bayesian optimization to systematically explore the hyperparameter space of a given machine learning model [11]. A probabilistic model, specifically Gaussian processes, generates a surrogate function to approximate the underlying objective function. This surrogate function facilitates the identification of promising regions within the hyperparameter space, thus enabling more efficient search and convergence. Furthermore, the acquisition function serves as a guide to balance the trade-off between exploration and exploitation in the search process. The BayesSearchCV algorithm's primary advantage lies in its ability to intelligently navigate the hyperparameter landscape, requiring fewer iterations to obtain optimal configurations.

GridSearchCV applies a comprehensive and systematic approach to hyperparameter tuning [12]. The cross-validated grid search technique entails exploring a pre-defined parameter space and evaluating various combinations of hyperparameters through k-fold cross-validation. By partitioning the dataset into "k" equally-sized subsets, the algorithm iteratively designates one subset as the validation set, while the remaining subsets function as the training set. The average performance metric is computed across all folds to determine the optimal hyperparameter configuration. The primary advantage of GridSearchCV lies in its intensive exploration of the parameter space, which facilitates the identification of the most effective hyperparameter combination, thus bolstering model performance and generalizability.

Optuna is an advanced, open-source hyperparameter optimization framework that offers a versatile and efficient method for tuning machine learning models [11]. Employing a sophisticated pruning strategy facilitates the swift identification of optimal hyperparameter configurations. Utilizing tree-structured Parzen estimators (TPE) for probabilistic modeling of hyperparameter search spaces, Optuna balances exploration and exploitation, thereby expediting the convergence to the global optimum. Moreover, the framework's modular design allows seamless integration with diverse machine-

learning libraries and offers an intuitive, user-friendly interface. Consequently, Optuna has garnered considerable attention in the scientific community as a powerful tool for streamlining model development and enhancing performance.

The rationale for combined Cohen-MCC metrics - The rationale for utilizing a combined metric of Cohen's Kappa and Matthew's Correlation Coefficient (MCC) in evaluating classification models stems from each metric's complementary strengths and weaknesses, ultimately resulting in a more robust and comprehensive model performance assessment. Cohen's Kappa is a popular statistical measure that evaluates the agreement between two raters or classifiers while accounting for the possibility of agreement occurring by chance. This metric ranges from -1 to 1, with 1 signifying perfect agreement, 0 indicating no better agreement than chance, and negative values representing disagreement. One of the critical advantages of Cohen's Kappa is its ability to account for class imbalance, making it suitable for scenarios with an uneven class distribution.

On the other hand, Matthew's Correlation Coefficient (MCC) is another widely used metric for evaluating binary classification models. It ranges from -1 to 1, with 1 representing perfect prediction, 0 indicating no better prediction than random, and -1 corresponding to a completely inverse prediction. MCC considers all elements of the confusion matrix (true positives, false positives, true negatives, and false negatives) and is robust in the presence of imbalanced classes. Combining these two metrics, one can capitalize on their strengths and offset their limitations. This dual metric approach provides a comprehensive evaluation of classification model performance that is less sensitive to class imbalance and encompasses the various aspects of model accuracy. As a result, the combined use of Cohen's Kappa and MCC offers a more informed and reliable assessment of classifier performance, facilitating better model selection and optimization for real-world applications.

NegLog2RMSL – A composite performance measure was devised by amalgamating the losses from training and testing predictions. Initially, this study evaluated disparities between training and testing accuracies, negative log-likelihood ratio, negative predictive value (NPV), precision, sensitivity, specificity, and the Brier score. Following this, the root mean square value (diff) of these differences was computed. The subsequent step determined the complements of accuracy, NPV, precision, sensitivity, and specificity (i.e., 1 - corresponding values) and integrated them with the negative likelihood ratio and Brier score. Afterward, the root mean square value (loss) was calculated as an encompassing performance indicator. Finally, the geometric mean of the discrepancy and loss values were computed and transformed to this outcome using the negative base-2 logarithm. This metric was denoted as NegLog2RMSL. Combining several performance metrics allows for a more comprehensive evaluation of a model's performance, capturing various aspects of prediction quality. By calculating the geometric mean of the root mean square values (diff and loss), NegLog2RMSL ensures a balanced assessment of overfitting, underfitting, and other biases in the model's performance. The conversion to negative log2 provides a uniform metric scaling, making comparing and interpreting values across different models and datasets easier. The NegLog2RMSL metric was designed for easy interpretation, providing a single value encapsulating multiple performance aspects. This simplifies the process of model comparison and selection.

References

1. Ianevski A., Giri A.K., Gautam P., Kononov A., Potdar S., Saarela J. et al. Prediction of drug combination effects with a minimal set of experiments, Nat Mach Intell 2019;1:568-577.
2. Ianevski A., Giri A.K., Aittokallio T. SynergyFinder 2.0: visual analytics of multi-drug combination synergies, Nucleic Acids Res 2020;48:W488-W493.
3. Chou T.C., Martin N. CompuSyn for Drug Combinations: PC Software and User's Guide: A Computer Program for Quantitation of Synergism and Antagonism in Drug Combinations, and the Determination of IC50 and ED50 and LD50 Values, ComboSyn Inc, Paramus, (NJ) 2005.
4. Chambers M.C., Maclean B., Burke R., Amodei D., Ruderman D.L., Neumann S. et al. A cross-platform toolkit for mass spectrometry and proteomics, Nat Biotechnol 2012;30:918-920.
5. Hakkinen J., Vincic G., Mansson O., Warell K., Levander F. The proteios software environment: an extensible multiuser platform for management and analysis of proteomics data, J Proteome Res 2009;8:3037-3043.
6. Kim S., Pevzner P.A. MS-GF+ makes progress towards a universal database search tool for proteomics, Nat Commun 2014;5:5277.
7. Teleman J., Chawade A., Sandin M., Levander F., Malmstrom J. Dinosaur: A Refined Open-Source Peptide MS Feature Detector, J Proteome Res 2016;15:2143-2151.
8. Braekeveldt N., von Stedingk K., Fransson S., Martinez-Monleon A., Lindgren D., Axelson H. et al. Patient-Derived Xenograft Models Reveal Intratumor Heterogeneity and Temporal Stability in Neuroblastoma, Cancer Res 2018;78:5958-5969.
9. Willforss J., Chawade A., Levander F. NormalyzerDE: Online Tool for Improved Normalization of Omics Expression Data and High-Sensitivity Differential Expression Analysis, J Proteome Res 2019;18:732-740.
10. Lemaître G., Nogueira F., Aridas C.K. Imbalanced-learn: A Python Toolbox to Tackle the Curse of Imbalanced Datasets in Machine Learning, Journal of Machine Learning Research 2017;18:1-5.

11. Akiba T., Sano S., Yanase T., Ohta T., Koyama M. Optuna: A Next-generation Hyperparameter Optimization Framework, Proceedings of the 25th ACM SIGKDD International Conference on Knowledge Discovery & Data Mining 2019;KDD 19:2623–2631.
12. Pedregosa F., Varoquaux G., Gramfort A., Michel V., Thirion B., Grisel O. et al. Scikit-learn: Machine Learning in Python, Journal of Machine Learning Research 2011;12:2825–2830.

Supplementary Figures

Supplementary Figure 1. Performance comparison of TabNet models with different sampling and hyperparameter search methods. (A-B) Detailed performance metrics for different sampling methods and hyperparameter searches. (C) A confusion matrix was created using test data.

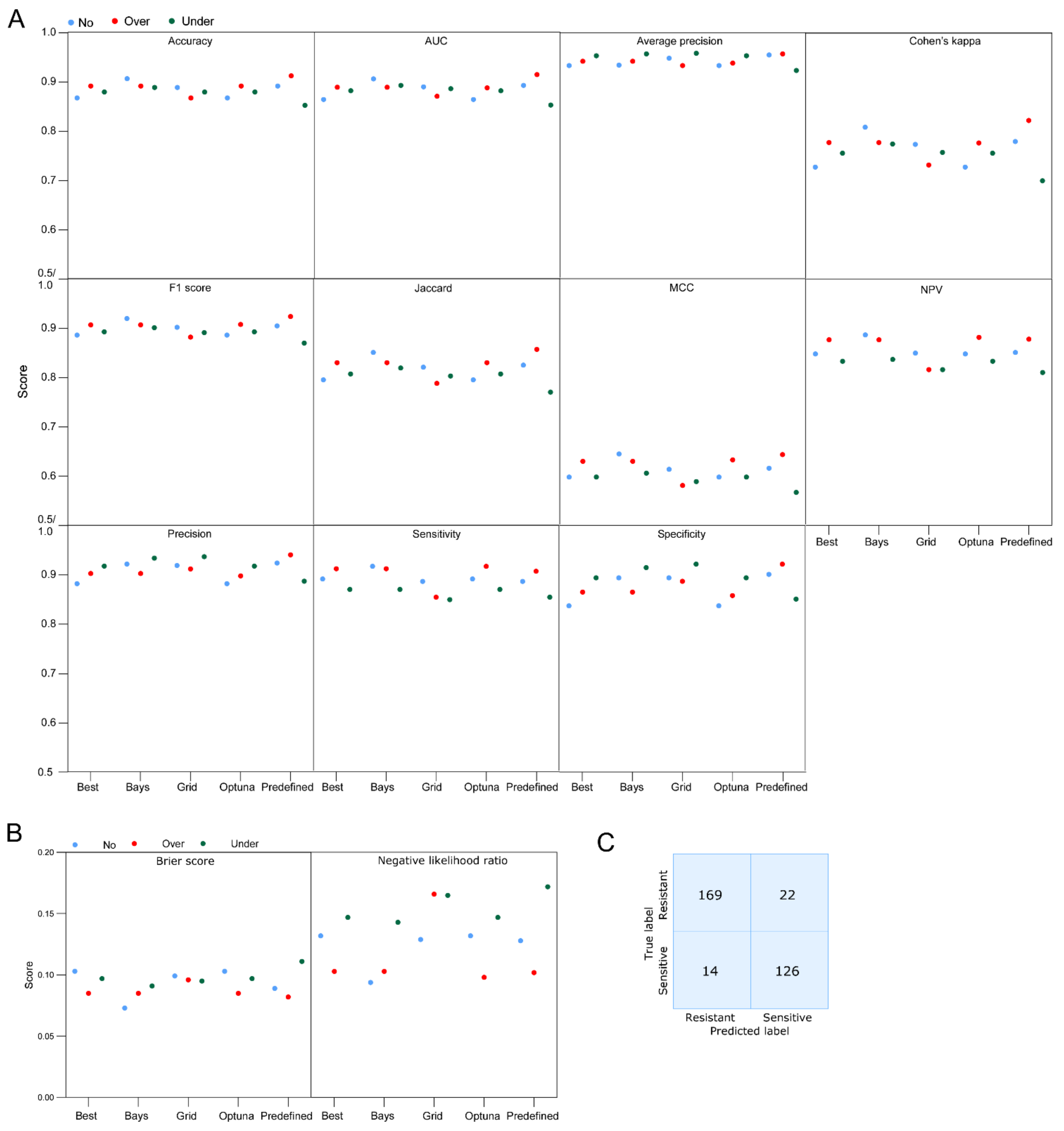

Supplementary Figure 2. The synergy between venetoclax and volasertib. (A) T-ALL cells were treated with different concentrations of venetoclax, volasertib, or a 1:1 combination. The full synergy matrix was generated using the DECREASE web app (http://decrease.fimm.fi/). The BLISS score was calculated by SynergyFinder (https://synergyfinder.fimm.fi/), and the combination index (CI) was calculated by ComboSyn (www.combosyn.com). (B) The synergy between venetoclax and other PKL-related inhibitors in T-ALL cells was measured in a similar fashion.

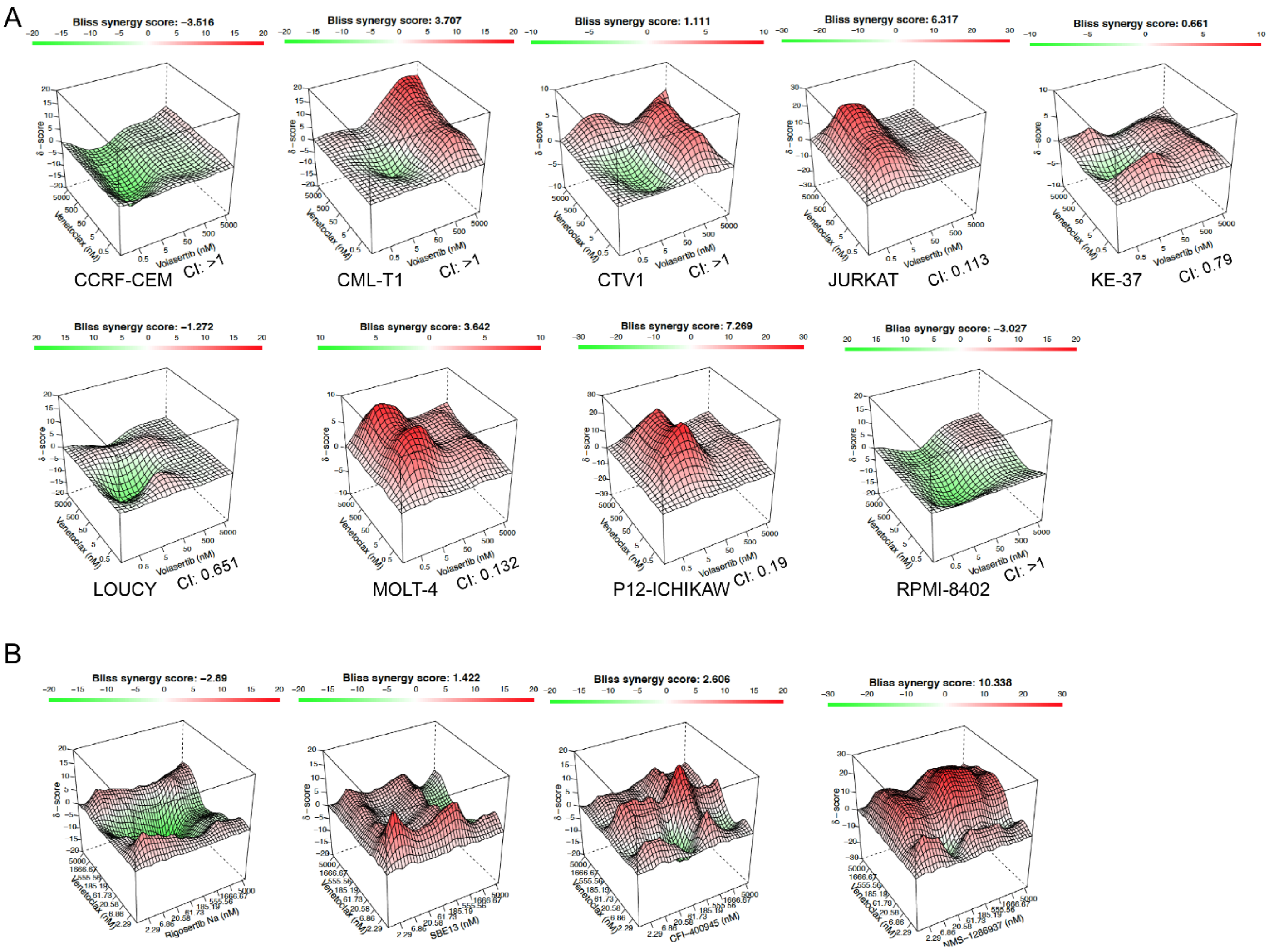

Supplementary Figure 3. Knockdown efficiency. (A) DND-41 cells were stability transfected with inducible PLK1 shRNA. Cells were then treated with doxycycline to induce PLK1 shRNA expression. Venetoclax was added to observe the synergistic effect. Knockdown efficiency was measured using western blotting. The PLK1 antibody repeatedly detected an additional band below the PLK1 band in cells overexpressing PLK1 shRNA. (B) DND41 cells were transfected with 100 nM siRNA. 4 hours after transfection, the media was changed to regular growth media and supplemented with vehicle or Venetoclax (5 µM). Aliquots were taken 24 h and 48 h after transfection to assess cell viability by CellTiterGlo. The right side shows knockdown efficacy. Samples for Taqman qRT-PCR were collected 48h after transfection.

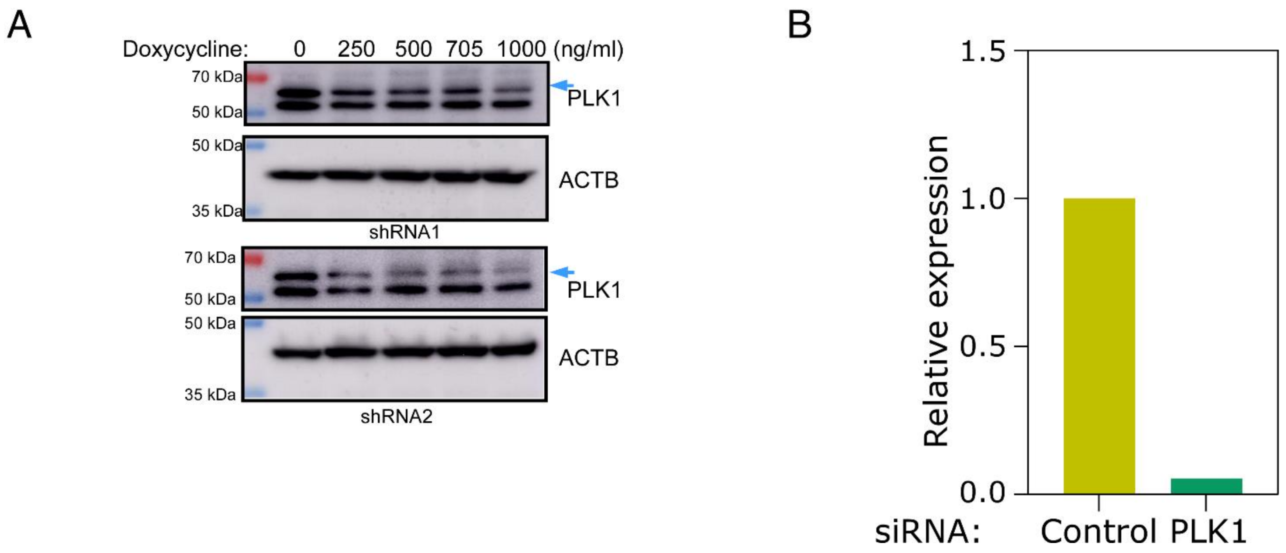

Supplementary Tables

Table 1. GSEA using HALLMARK dataset.

NAME	ES	NES	NOM p-val	FDR q-val
HALLMARK_TNFA_SIGNALING_VIA_NFKB	0.474	2.344	0.000	0.000
HALLMARK_G2M_CHECKPOINT	0.420	2.091	0.000	0.000
HALLMARK_MITOTIC_SPINDLE	0.418	2.071	0.000	0.000
HALLMARK_TGF_BETA_SIGNALING	0.506	2.016	0.000	0.000
HALLMARK_KRAS_SIGNALING_UP	0.407	1.925	0.000	0.000
HALLMARK_HEME_METABOLISM	0.366	1.811	0.000	0.002
HALLMARK_INFLAMMATORY_RESPONSE	0.377	1.833	0.000	0.002
HALLMARK_UV_RESPONSE_DN	0.382	1.787	0.000	0.002
HALLMARK_PROTEIN_SECRETION	0.374	1.647	0.002	0.010
HALLMARK_ANDROGEN_RESPONSE	0.357	1.571	0.005	0.019
HALLMARK_E2F_TARGETS	0.308	1.528	0.001	0.028
HALLMARK_APOPTOSIS	0.298	1.423	0.016	0.066
HALLMARK_HEDGEHOG_SIGNALING	0.409	1.409	0.073	0.070
HALLMARK_COMPLEMENT	0.273	1.333	0.035	0.127

Table 2. GSEA using KEGG dataset.

NAME	ES	NES	NOM p-val	FDR q-val
KEGG_TGF_BETA_SIGNALING_PATHWAY	0.489	1.994	0.000	0.012
KEGG_NOD_LIKE_RECEPTOR_SIGNALING_PATHWAY	0.454	1.833	0.000	0.051
KEGG_CELL_CYCLE	0.373	1.729	0.000	0.079
KEGG_ADHERENS_JUNCTION	0.420	1.744	0.002	0.091
KEGG_P53_SIGNALING_PATHWAY	0.411	1.691	0.005	0.092
KEGG_GNRH_SIGNALING_PATHWAY	0.374	1.615	0.005	0.097
KEGG_CHRONIC_MYELOID_LEUKEMIA	0.393	1.660	0.000	0.102
KEGG_LONG_TERM_DEPRESSION	0.427	1.618	0.009	0.104
KEGG_NEUROTROPHIN_SIGNALING_PATHWAY	0.344	1.589	0.007	0.110
KEGG_JAK_STAT_SIGNALING_PATHWAY	0.349	1.581	0.004	0.110
KEGG_ALDOSTERONE_REGULATED_SODIUM_REABSORPTION	0.453	1.569	0.022	0.113
KEGG_T_CELL_RECEPTOR_SIGNALING_PATHWAY	0.362	1.623	0.000	0.113
KEGG_GLIOMA	0.383	1.550	0.018	0.114
KEGG_ERBB_SIGNALING_PATHWAY	0.361	1.557	0.004	0.116
KEGG_COLORECTAL_CANCER	0.399	1.625	0.003	0.127
KEGG_FC_EPSILON_RI_SIGNALING_PATHWAY	0.363	1.505	0.019	0.127
KEGG_PROSTATE_CANCER	0.352	1.527	0.010	0.128
KEGG_SMALL_CELL_LUNG_CANCER	0.361	1.508	0.017	0.132
KEGG_ENDOMETRIAL_CANCER	0.383	1.478	0.030	0.137
KEGG_RENAL_CELL_CARCINOMA	0.362	1.508	0.020	0.139
KEGG_TIGHT_JUNCTION	0.337	1.480	0.027	0.142
KEGG_GRAFT_VERSUS_HOST_DISEASE	0.413	1.484	0.025	0.144
KEGG_PATHWAYS_IN_CANCER	0.286	1.459	0.003	0.152
KEGG_PANCREATIC_CANCER	0.346	1.453	0.032	0.152
KEGG_ARRHYTHMOGENIC_RIGHT_VENTRICULAR_CARDIOMYOPATHY_ARVC	0.363	1.426	0.042	0.177
KEGG_CHEMOKINE_SIGNALING_PATHWAY	0.295	1.412	0.020	0.189
KEGG_PROGESTERONE_MEDIATED_OOCYTE_MATURATION	0.329	1.400	0.040	0.190
KEGG_INSULIN_SIGNALING_PATHWAY	0.291	1.365	0.042	0.192
KEGG_UBIQUITIN_MEDIATED_PROTEOLYSIS	0.298	1.403	0.018	0.193
KEGG_WNT_SIGNALING_PATHWAY	0.299	1.377	0.026	0.195

Table 3. GSEA using PID dataset.

NAME	ES	NES	NOM p-val	FDR q-val
PID_ERBB4_PATHWAY	0.621	2.153	0.000	0.002
PID_FOXO_PATHWAY	0.538	2.105	0.000	0.002
PID_E2F_PATHWAY	0.451	1.899	0.000	0.018
PID_TGFBR_PATHWAY	0.482	1.918	0.000	0.018
PID_SMAD2_3NUCLEAR_PATHWAY	0.448	1.922	0.000	0.022
PID_AR_TF_PATHWAY	0.480	1.865	0.000	0.024
PID_FAK_PATHWAY	0.482	1.935	0.000	0.026
PID_NFKAPPAB_CANONICAL_PATHWAY	0.513	1.698	0.013	0.090
PID_TCPTP_PATHWAY	0.462	1.705	0.003	0.091
PID_ER_NONGENOMIC_PATHWAY	0.445	1.637	0.013	0.092
PID_IL6_7_PATHWAY	0.446	1.712	0.005	0.094
PID_HES_HEY_PATHWAY	0.438	1.629	0.011	0.094
PID_IL4_2PATHWAY	0.420	1.654	0.005	0.094
PID_NFKAPPAB_ATYPICAL_PATHWAY	0.550	1.638	0.016	0.096
PID_ERBB1_RECEPTOR_PROXIMAL_PATHWAY	0.459	1.656	0.008	0.098
PID_ERBB1_INTERNALIZATION_PATHWAY	0.462	1.720	0.002	0.098
PID_ATF2_PATHWAY	0.423	1.681	0.005	0.099
PID_TELOMERASE_PATHWAY	0.397	1.671	0.002	0.099
PID_ANGIOPOIETIN_RECEPTOR_PATHWAY	0.429	1.638	0.005	0.102
PID_KIT_PATHWAY	0.397	1.588	0.013	0.103
PID_PLK1_PATHWAY	0.416	1.593	0.013	0.103
PID_AR_PATHWAY	0.382	1.577	0.009	0.104
PID_BARD1_PATHWAY	0.477	1.657	0.008	0.104
PID_FRA_PATHWAY	0.448	1.612	0.012	0.105
PID_RETINOIC_ACID_PATHWAY	0.461	1.596	0.020	0.105
PID_LYMPH_ANGIOGENESIS_PATHWAY	0.463	1.569	0.033	0.105
PID_BMP_PATHWAY	0.447	1.578	0.018	0.107
PID_FAS_PATHWAY	0.426	1.569	0.024	0.108
PID_EPO_PATHWAY	0.458	1.597	0.013	0.108
PID_SHP2_PATHWAY	0.409	1.599	0.014	0.112
PID_CMYB_PATHWAY	0.360	1.548	0.006	0.121
PID_CIRCADIAN_PATHWAY	0.525	1.538	0.032	0.121
PID_HEDGEHOG_GLI_PATHWAY	0.405	1.542	0.016	0.123
PID_AP1_PATHWAY	0.372	1.530	0.022	0.126
PID_TNF_PATHWAY	0.398	1.520	0.019	0.129
PID_P38_ALPHA_BETA_DOWNSTREAM_PATHWAY	0.416	1.523	0.031	0.129
PID_FANCONI_PATHWAY	0.394	1.510	0.031	0.132
PID_RB_1PATHWAY	0.371	1.513	0.022	0.133
PID_BETA_CATENIN_NUC_PATHWAY	0.354	1.477	0.026	0.159
PID_GMCSF_PATHWAY	0.412	1.477	0.045	0.163
PID_REELIN_PATHWAY	0.446	1.469	0.045	0.165
PID_P53_REGULATION_PATHWAY	0.361	1.453	0.041	0.177
PID_IL2_STAT5_PATHWAY	0.414	1.454	0.048	0.180

Table 4. GSEA using GOBP dataset.

NAME	ES	NES	NOM p-val	FDR q-val
GOBP_POSITIVE_REGULATION_OF_MRNA_METABOLIC_PROCESS	0.464	2.159	0.000	0.009
GOBP_PHOSPHATIDYLINOSITOL_3_PHOSPHATE_BIOSYNTHETIC_PROCESS	0.662	2.112	0.000	0.009
GOBP_REGULATION_OF_CENTRIOLE_REPLICATION	0.654	2.116	0.002	0.010
GOBP_REGULATION_OF_NUCLEAR_TRANSCRIBED_MRNA_CATABOLIC_PROCESS_DEADENYLATION_DEPENDENT_DECAY	0.635	2.126	0.000	0.010
GOBP_CENTRIOLE_ASSEMBLY	0.567	2.178	0.000	0.010
GOBP_REGULATION_OF_MRNA_METABOLIC_PROCESS	0.421	2.162	0.000	0.011
GOBP_REGULATION_OF_MRNA_PROCESSING	0.475	2.203	0.000	0.012
GOBP_MAINTENANCE_OF_CELL_NUMBER	0.431	2.087	0.000	0.013
GOBP_ALTERNATIVE_MRNA_SPLICING_VIA_SPLICEOSOME	0.491	2.056	0.000	0.013
GOBP_NLS_BEARING_PROTEIN_IMPORT_INTO_NUCLEUS	0.675	2.080	0.000	0.013
GOBP_CENTROSOME_DUPLICATION	0.494	2.060	0.000	0.013
GOBP_REGULATION_OF_RNA_SPLICING	0.422	2.068	0.000	0.014
GOBP_RNA_DESTABILIZATION	0.491	2.224	0.000	0.016
GOBP_HISTONE_H3_K9_MODIFICATION	0.523	2.031	0.000	0.018
GOBP_NUCLEAR_TRANSCRIBED_MRNA_CATABOLIC_PROCESS_DEADENYLATION_DEPENDENT_DECAY	0.483	2.033	0.000	0.018
GOBP_MICROTUBULE_ORGANIZING_CENTER_ORGANIZATION	0.430	2.019	0.000	0.020
GOBP_RNA_EXPORT_FROM_NUCLEUS	0.456	2.008	0.000	0.021
GOBP_POSITIVE_REGULATION_OF_CELL_CYCLE_PROCESS	0.395	2.012	0.000	0.021
GOBP_REGULATION_OF_ALTERNATIVE_MRNA_SPLICING_VIA_SPLICEOSOME	0.502	1.995	0.000	0.021
GOBP_REGULATION_OF_MRNA_CATABOLIC_PROCESS	0.412	1.982	0.000	0.021
GOBP_RNA_POLYADENYLATION	0.530	1.990	0.000	0.021
GOBP_3_UTR_MEDIATED_MRNA_DESTABILIZATION	0.686	2.000	0.000	0.022
GOBP_MRNA_PROCESSING	0.369	1.982	0.000	0.022
GOBP_POSITIVE_REGULATION_OF_HISTONE_METHYLATION	0.515	1.995	0.000	0.022
GOBP_NEGATIVE_REGULATION_OF_CELLULAR_AMIDE_METABOLIC_PROCESS	0.406	1.983	0.000	0.023
GOBP_POSITIVE_REGULATION_OF_CELL_CYCLE	0.378	1.962	0.000	0.024
GOBP_POSITIVE_REGULATION_OF_ORGANELLE_ASSEMBLY	0.455	1.957	0.000	0.024
GOBP_MITOTIC_G2_DNA_DAMAGE_CHECKPOINT_SIGNALING	0.543	1.970	0.000	0.024
GOBP_POSITIVE_REGULATION_OF_CYTOKINESIS	0.554	1.972	0.002	0.024
GOBP_CELL_CYCLE_DNA_REPLICATION	0.510	1.963	0.000	0.024
GOBP_DNA_TEMPLATED_DNA_REPLICATION_MAINTENANCE_OF_FIDELITY	0.488	1.957	0.000	0.025
GOBP_MRNA_3_END_PROCESSING	0.486	1.951	0.000	0.025
GOBP_MRNA_TRANSPORT	0.418	1.949	0.000	0.025
GOBP_HISTONE_MODIFICATION	0.363	1.963	0.000	0.025
GOBP_REGULATION_OF_MRNA_SPLICING_VIA_SPLICEOSOME	0.441	1.964	0.000	0.025
GOBP_PROTEIN_MODIFICATION_BY_SMALL_PROTEIN_REMOVAL	0.404	1.951	0.000	0.025
GOBP_NUCLEAR_TRANSCRIBED_MRNA_CATABOLIC_PROCESS	0.417	1.945	0.000	0.026
GOBP_POSITIVE_REGULATION_OF_GLUCOSE_TRANSMEMBRANE_TRANSPORT	0.535	1.942	0.000	0.026
GOBP_RESPONSE_TO_MANGANESE_ION	0.625	1.933	0.000	0.028
GOBP_GLOMERULAR_EPITHELIAL_CELL_DIFFERENTIATION	0.664	1.930	0.000	0.029
GOBP_MRNA_EXPORT_FROM_NUCLEUS	0.465	1.927	0.000	0.030

GOBP_GENE_SILENCING_BY_RNA	0.429	1.923	0.000	0.030
GOBP_MUSCLE_HYPERTROPHY_IN_RESPONSE_TO_STRESS	0.662	1.917	0.000	0.032
GOBP_DNA_INTEGRITY_CHECKPOINT_SIGNALING	0.406	1.909	0.000	0.034
GOBP_REGULATION_OF_HISTONE_METHYLATION	0.444	1.911	0.000	0.034
GOBP_MICROTUBULE_ORGANIZING_CENTER_LOCALIZATION	0.532	1.904	0.002	0.035
GOBP_REGULATION_OF_NUCLEOCYTOPLASMIC_TRANSPORT	0.424	1.900	0.000	0.035
GOBP_MRNA_SPLICE_SITE_SELECTION	0.543	1.901	0.002	0.036
GOBP_PROTEIN_K48_LINKED_DEUBIQUITINATION	0.537	1.890	0.002	0.036
GOBP_BONE_MINERALIZATION	0.426	1.896	0.000	0.036
GOBP_REGULATION_OF_CHROMATIN_ORGANIZATION	0.494	1.893	0.000	0.036
GOBP_NEGATIVE_REGULATION_OF_GENE_EXPRESSION_EPIGENETIC	0.441	1.894	0.002	0.036
GOBP_RNA_SPLICING	0.351	1.890	0.000	0.037
GOBP_NUCLEAR_TRANSPORT	0.361	1.881	0.000	0.037
GOBP_HETEROCHROMATIN_ORGANIZATION	0.436	1.882	0.000	0.037
GOBP_MRNA_CATABOLIC_PROCESS	0.373	1.884	0.000	0.038
GOBP_ESTABLISHMENT_OF_RNA_LOCALIZATION	0.393	1.883	0.000	0.038
GOBP_REGULATION_OF_MRNA_3_END_PROCESSING	0.583	1.873	0.002	0.040
GOBP_HISTONE_MONOUBIQUITINATION	0.516	1.867	0.003	0.042
GOBP_OVULATION	0.646	1.865	0.000	0.042
GOBP_FEMALE_GAMETE_GENERATION	0.403	1.861	0.000	0.043
GOBP_REGULATION_OF_CYTOPLASMIC_TRANSLATION	0.564	1.858	0.002	0.043
GOBP_POSITIVE_REGULATION_OF_CYCLIN_DEPENDENT_PROTEIN_KINASE_ACTIVITY	0.536	1.860	0.000	0.043
GOBP_BLASTOCYST_GROWTH	0.595	1.862	0.005	0.043
GOBP_SUBSTRATE_DEPENDENT_CELL_MIGRATION	0.582	1.850	0.002	0.044
GOBP_ENDOPLASMIC_RETICULUM_TUBULAR_NETWORK_ORGANIZATION	0.620	1.848	0.002	0.044
GOBP_NUCLEOTIDE_BINDING_DOMAIN_LEUCINE_RICH_REPEAT_CONTAINING_RECEPTOR_SIGNALING_PATHWAY	0.560	1.852	0.000	0.044
GOBP_PEPTIDYL_LYSINE_TRIMETHYLATION	0.463	1.846	0.002	0.045
GOBP_REPLICATION_FORK_PROCESSING	0.484	1.853	0.002	0.045
GOBP_DETECTION_OF_OTHER_ORGANISM	0.617	1.849	0.005	0.045
GOBP_REGULATION_OF_HETEROCHROMATIN_FORMATION	0.549	1.843	0.000	0.046
GOBP_HISTONE_UBIQUITINATION	0.469	1.835	0.000	0.046
GOBP_WOUND_HEALING_SPREADING_OF_EPIDERMAL_CELLS	0.621	1.837	0.002	0.047
GOBP_NEUROBLAST_PROLIFERATION	0.484	1.839	0.002	0.047
GOBP_CHONDROCYTE_DEVELOPMENT	0.554	1.836	0.000	0.047
GOBP_MITOTIC_G2_M_TRANSITION_CHECKPOINT	0.469	1.837	0.002	0.047
GOBP_NEURONAL_STEM_CELL_POPULATION_MAINTENANCE	0.604	1.832	0.002	0.047
GOBP_HISTONE_MRNA_CATABOLIC_PROCESS	0.635	1.832	0.007	0.048
GOBP_PEPTIDYL_LYSINE_METHYLATION	0.392	1.829	0.000	0.048
GOBP_REGULATION_OF_BONE_MINERALIZATION	0.450	1.827	0.000	0.048
GOBP_SIGNAL_TRANSDUCTION_IN_RESPONSE_TO_DNA_DAMAGE	0.366	1.821	0.000	0.051
GOBP_REGULATION_OF_RAC_PROTEIN_SIGNAL_TRANSDUCTION	0.575	1.815	0.007	0.051
GOBP_PROTEIN_ACETYLATION	0.364	1.818	0.000	0.051
GOBP_NUCLEAR_EXPORT	0.377	1.819	0.000	0.051
GOBP_NEGATIVE_REGULATION_OF_MRNA_METABOLIC_PROCESS	0.421	1.815	0.000	0.051
GOBP_NEGATIVE_REGULATION_OF_CELL_CYCLE_G2_M_PHASE_TRANSITION	0.436	1.816	0.000	0.052
GOBP_POSITIVE_REGULATION_OF_GLUCOSE_IMPORT	0.522	1.811	0.005	0.053
GOBP_REGULATION_OF_CYTOKINESIS	0.421	1.810	0.002	0.053

GOBP_OOGENESIS	0.419	1.803	0.002	0.055
GOBP_REGULATION_OF_BIOMINERALIZATION	0.421	1.805	0.000	0.055
GOBP_HISTONE_H3_K4_METHYLATION	0.428	1.803	0.000	0.055
GOBP_CELLULAR_COMPONENT_ASSEMBLY_INVOLVED_IN_MORPHOGENESIS	0.420	1.804	0.002	0.055
GOBP_CELL_CYCLE_G1_S_PHASE_TRANSITION	0.354	1.792	0.000	0.060
GOBP_POSITIVE_REGULATION_OF_CHROMATIN_ORGANIZATION	0.589	1.793	0.004	0.060
GOBP_REGULATION_OF_CELLULAR_RESPONSE_TO_HEAT	0.598	1.777	0.002	0.063
GOBP_CIRCADIAN_RHYTHM	0.366	1.784	0.000	0.063
GOBP_NUCLEAR_TRANSCRIBED_MRNA_POLY_A_TAIL_SHORTENING	0.510	1.786	0.003	0.064
GOBP_HISTONE_H3_K4_TRIMETHYLATION	0.560	1.787	0.003	0.064
GOBP_HISTONE_METHYLATION	0.368	1.777	0.000	0.064
GOBP_RHYTHMIC_PROCESS	0.352	1.778	0.000	0.064
GOBP_PHOSPHOLIPID_DEPHOSPHORYLATION	0.478	1.773	0.003	0.064
GOBP_RNA_STABILIZATION	0.441	1.779	0.002	0.064
GOBP_NEGATIVE_REGULATION_OF_RNA_CATABOLIC_PROCESS	0.431	1.784	0.000	0.064
GOBP_NEGATIVE_REGULATION_OF_DNA_REPLICATION	0.515	1.771	0.003	0.064
GOBP_REGULATION_OF_T_CELL_DIFFERENTIATION_IN_THYMUS	0.538	1.774	0.005	0.064
GOBP_IMPORT_INTO_NUCLEUS	0.370	1.779	0.000	0.064
GOBP_POSITIVE_REGULATION_OF_DNA_REPLICATION	0.479	1.771	0.000	0.065
GOBP_NEGATIVE_REGULATION_OF_CELL_CYCLE	0.336	1.774	0.000	0.065
GOBP_HISTONE_H2A_UBIQUITINATION	0.501	1.769	0.003	0.065
GOBP_RNA_SPLICING_VIA_TRANSESTERIFICATION_REACTIONS	0.341	1.780	0.000	0.065
GOBP_CELL_CYCLE_PHASE_TRANSITION	0.322	1.771	0.000	0.065
GOBP_REGULATION_OF_CENTROSOME_CYCLE	0.448	1.780	0.002	0.065
GOBP_POSITIVE_REGULATION_OF_VASCULAR_ENDOTHELIAL_GROWTH_FACTOR_PRODUCTION	0.544	1.760	0.003	0.068
GOBP_CELL_DIFFERENTIATION_INVOLVED_IN_KIDNEY_DEVELOPMENT	0.473	1.760	0.003	0.069
GOBP_REGULATION_OF_STEM_CELL_POPULATION_MAINTENANCE	0.428	1.762	0.003	0.069
GOBP_REGULATION_OF_ORGANELLE_ASSEMBLY	0.350	1.761	0.001	0.069
GOBP_REGULATION_OF_CELLULAR_RESPONSE_TO_TRANSFORMING_GROWTH_FACTOR_BETA_STIMULUS	0.380	1.757	0.000	0.069
GOBP_DNA_REPLICATION	0.339	1.758	0.000	0.069
GOBP_PALLIUM_DEVELOPMENT	0.377	1.752	0.000	0.070
GOBP_HISTONE_H3_K9_ACETYLATION	0.574	1.753	0.003	0.070
GOBP_EMBRYONIC_PLACENTA_DEVELOPMENT	0.424	1.751	0.002	0.070
GOBP_SMALL_REGULATORY_NCRNA_PROCESSING	0.434	1.754	0.002	0.070
GOBP_NEGATIVE_REGULATION_OF_SMALL_GTPASE_MEDIATED_SIGNAL_TRANSDUCTION	0.444	1.750	0.005	0.070
GOBP_NEGATIVE_REGULATION_OF_CELL_CYCLE_PROCESS	0.341	1.754	0.000	0.070
GOBP_NEGATIVE_REGULATION_OF_STEM_CELL_POPULATION_MAINTENANCE	0.542	1.747	0.005	0.070
GOBP_RESPONSE_TO_MUSCLE_STRETCH	0.541	1.742	0.003	0.071
GOBP_RESPONSE_TO_TRANSFORMING_GROWTH_FACTOR_BETA	0.344	1.748	0.000	0.071
GOBP_MITOTIC_CELL_CYCLE_PHASE_TRANSITION	0.322	1.744	0.000	0.071
GOBP_INNATE_IMMUNE_RESPONSE_ACTIVATING_SIGNAL_TRANSDUCTION	0.517	1.744	0.005	0.071
GOBP_REGULATION_OF_HISTONE_MODIFICATION	0.359	1.743	0.000	0.071
GOBP_MUSCLE_CELL_PROLIFERATION	0.362	1.745	0.000	0.071
GOBP_NEGATIVE_REGULATION_OF_BMP_SIGNALING_PATHWAY	0.485	1.748	0.000	0.071
GOBP_FOREBRAIN_DEVELOPMENT	0.340	1.745	0.000	0.072
GOBP_RNA_LOCALIZATION	0.350	1.739	0.000	0.072

GOBP_POSITIVE_REGULATION_OF_NUCLEOCYTOPLASMIC_TRANSPORT	0.426	1.735	0.006	0.074
GOBP_RAS_PROTEIN_SIGNAL_TRANSDUCTION	0.326	1.736	0.000	0.074
GOBP_PEPTIDYL_THREONINE_MODIFICATION	0.382	1.730	0.001	0.077
GOBP_HISTONE_H3_ACETYLATION	0.396	1.731	0.000	0.077
GOBP_KERATINOCYTE_MIGRATION	0.591	1.726	0.005	0.078
GOBP_REGULATION_OF_DNA_METABOLIC_PROCESS	0.316	1.726	0.000	0.079
GOBP_N_TERMINAL_PROTEIN_AMINO_ACID_MODIFICATION	0.503	1.727	0.005	0.079
GOBP_MRNA_TRANSCRIPTION	0.444	1.724	0.003	0.079
GOBP_PEROXISOME_PROLIFERATOR_ACTIVATED_RECEPTOR_SIGNALING_PATHWAY	0.592	1.721	0.005	0.081
GOBP_BIOMINERALIZATION	0.375	1.720	0.003	0.081
GOBP_DNA_METHYLATION_DEPENDENT_HETEROCHROMATIN_FORMATION	0.519	1.718	0.013	0.082
GOBP_PROTEIN_ACYLATION	0.333	1.715	0.000	0.083
GOBP_MITOTIC_DNA_INTEGRITY_CHECKPOINT_SIGNALING	0.397	1.715	0.002	0.083
GOBP_NATURAL_KILLER_CELL_DIFFERENTIATION	0.534	1.716	0.009	0.083
GOBP_REGULATION_OF_CYCLIN_DEPENDENT_PROTEIN_KINASE_ACTIVITY	0.386	1.713	0.000	0.083
GOBP_NEGATIVE_REGULATION_OF_MITOTIC_CELL_CYCLE	0.338	1.713	0.000	0.083
GOBP_CIRCADIAN_REGULATION_OF_GENE_EXPRESSION	0.418	1.714	0.005	0.083
GOBP_PEPTIDYL_LYSINE_ACETYLATION	0.346	1.710	0.000	0.084
GOBP_NEGATIVE_REGULATION_OF_MITOTIC_CELL_CYCLE_PHASE_TRANSITION	0.348	1.708	0.000	0.085
GOBP_NUCLEOTIDE_BINDING_OLIGOMERIZATION_DOMAIN_CONTAINING_2_SIGNALING_PATHWAY	0.579	1.707	0.005	0.086
GOBP_VASCULAR_ENDOTHELIAL_GROWTH_FACTOR_PRODUCTION	0.491	1.704	0.012	0.087
GOBP_NEGATIVE_REGULATION_OF_EPITHELIAL_CELL_APOPTOTIC_PROCESS	0.462	1.704	0.003	0.087
GOBP_ADHERENS_JUNCTION_ORGANIZATION	0.468	1.702	0.008	0.088
GOBP_HISTONE_H2A_MONOUBIQUITINATION	0.524	1.701	0.003	0.089
GOBP_PHOSPHATIDYLINOSITOL_DEPHOSPHORYLATION	0.496	1.700	0.008	0.089
GOBP_DNA_TEMPLATED_DNA_REPLICATION	0.348	1.697	0.000	0.089
GOBP_REGULATION_OF_PLASMA_MEMBRANE_ORGANIZATION	0.568	1.690	0.007	0.089
GOBP_PRIMARY_MIRNA_PROCESSING	0.572	1.698	0.013	0.090
GOBP_MUSCLE_HYPERTROPHY	0.416	1.697	0.005	0.090
GOBP_NUCLEUS_ORGANIZATION	0.359	1.692	0.000	0.090
GOBP_CELL_DIFFERENTIATION_INVOLVED_IN_EMBRYONIC_PLACENTA_DEVELOPMENT	0.541	1.690	0.015	0.090
GOBP_3_UTR_MEDIATED_MRNA_STABILIZATION	0.554	1.691	0.013	0.090
GOBP_RAC_PROTEIN_SIGNAL_TRANSDUCTION	0.458	1.692	0.003	0.090
GOBP_DNA_TEMPLATED_TRANSCRIPTION_ELONGATION	0.331	1.692	0.000	0.090
GOBP_POSITIVE_REGULATION_OF_CELL_SUBSTRATE_JUNCTION_ORGANIZATION	0.513	1.688	0.007	0.091
GOBP_RESPONSE_TO_LECTIN	0.549	1.692	0.007	0.091
GOBP_TRANSFORMING_GROWTH_FACTOR_BETA_RECEPTOR_SIGNALING_PATHWAY	0.348	1.694	0.000	0.091
GOBP_DOSAGE_COMPENSATION	0.485	1.692	0.020	0.091
GOBP_SISTER_CHROMATID_COHESION	0.428	1.686	0.005	0.091
GOBP_POLY_A_PLUS_MRNA_EXPORT_FROM_NUCLEUS	0.533	1.685	0.018	0.091
GOBP_MALE_MEIOTIC_NUCLEAR_DIVISION	0.475	1.693	0.010	0.092
GOBP_PLASMA_MEMBRANE_TUBULATION	0.541	1.693	0.007	0.092
GOBP_KERATINOCYTE_PROLIFERATION	0.478	1.686	0.007	0.092
GOBP_SPLICEOSOMAL_COMPLEX_ASSEMBLY	0.394	1.682	0.002	0.092
GOBP_STRIATED_MUSCLE_ADAPTATION	0.485	1.683	0.008	0.092
GOBP_OVULATION_CYCLE_PROCESS	0.466	1.679	0.012	0.092

GOBP_PHOSPHATIDYLINOSITOL_PHOSPHATE_BIOSYNTHETIC_PROCESS	0.407	1.681	0.002	0.092
GOBP_PEPTIDYL_LYSINE_MODIFICATION	0.314	1.680	0.000	0.092
GOBP_NEGATIVE_REGULATION_OF_RHO_PROTEIN_SIGNAL_TRANSDUCTION	0.540	1.683	0.019	0.093
GOBP_CELL_CYCLE_G2_M_PHASE_TRANSITION	0.350	1.680	0.000	0.093
GOBP_RNA_CATABOLIC_PROCESS	0.331	1.680	0.000	0.093
GOBP_REGULATION_OF_POTASSIUM_ION_TRANSMEMBRANE_TRANSPORTER_ACTIVITY	0.447	1.677	0.007	0.093
GOBP_REGULATION_OF_PROTEIN_IMPORT_INTO_NUCLEUS	0.424	1.674	0.003	0.093
GOBP_PEPTIDYL_LYSINE_DIMETHYLATION	0.509	1.674	0.010	0.093
GOBP_CHONDROCYTE_DIFFERENTIATION	0.385	1.677	0.006	0.093
GOBP_LENS_MORPHOGENESIS_IN_CAMERA_TYPE_EYE	0.568	1.676	0.010	0.094
GOBP_MIRNA_METABOLIC_PROCESS	0.388	1.675	0.003	0.094
GOBP_N_TERMINAL_PROTEIN_AMINO_ACID_ACETYLATION	0.560	1.674	0.016	0.094
GOBP_INSULIN_RECEPTOR_SIGNALING_PATHWAY	0.371	1.674	0.003	0.094
GOBP_CELLULAR_RESPONSE_TO_PEPTIDE_HORMONE_STIMULUS	0.328	1.671	0.000	0.095
GOBP_REGULATION_OF_DNA_REPLICATION	0.353	1.671	0.001	0.095
GOBP_FACULTATIVE_HETEROCHROMATIN_FORMATION	0.471	1.668	0.008	0.096
GOBP_MODULATION_OF_PROCESS_OF_ANOTHER_ORGANISM	0.557	1.669	0.012	0.096
GOBP_POSITIVE_REGULATION_OF_CELL_CYCLE_PHASE_TRANSITION	0.368	1.668	0.004	0.096
GOBP_PEPTIDYL_SERINE_MODIFICATION	0.326	1.663	0.000	0.096
GOBP_POSITIVE_REGULATION_OF_ERBB_SIGNALING_PATHWAY	0.460	1.668	0.008	0.096
GOBP_REGULATION_OF_RAS_PROTEIN_SIGNAL_TRANSDUCTION	0.345	1.662	0.000	0.096
GOBP_POSITIVE_REGULATION_OF_HISTONE_MODIFICATION	0.377	1.663	0.000	0.096
GOBP_POSITIVE_REGULATION_OF_NITRIC_OXIDE_SYNTHASE_BIOSYNTHETIC_PROCESS	0.552	1.663	0.016	0.097
GOBP_REGULATION_OF_EXTRINSIC_APOPTOTIC_SIGNALING_PATHWAY_VIA_DEATH_DOMAIN_RECEPTORS	0.444	1.664	0.005	0.097
GOBP_PLACENTA_DEVELOPMENT	0.364	1.666	0.001	0.097
GOBP_POSITIVE_REGULATION_OF_CELLULAR_CATABOLIC_PROCESS	0.310	1.665	0.000	0.097
GOBP_RESPONSE_TO_FUNGUS	0.427	1.664	0.006	0.097
GOBP_DEFENSE_RESPONSE_TO_FUNGUS	0.450	1.661	0.003	0.097
GOBP_REGULATION_OF_CELL_CYCLE_PHASE_TRANSITION	0.309	1.664	0.000	0.097
GOBP_REGULATION_OF_CELL_CYCLE_G2_M_PHASE_TRANSITION	0.367	1.658	0.001	0.097
GOBP_HISTONE_H3_K9_METHYLATION	0.476	1.658	0.011	0.098
GOBP_CELL_CELL_JUNCTION_ORGANIZATION	0.351	1.656	0.000	0.098
GOBP_PROTEIN_K63_LINKED_DEUBIQUITINATION	0.456	1.658	0.003	0.098
GOBP_MRNA_METHYLATION	0.563	1.656	0.012	0.098
GOBP_EMBRYONIC_EYE_MORPHOGENESIS	0.509	1.659	0.015	0.098
GOBP_REGULATION_OF_CELL_SUBSTRATE_JUNCTION_ORGANIZATION	0.411	1.656	0.008	0.098
GOBP_CELL_CYCLE_CHECKPOINT_SIGNALING	0.339	1.651	0.000	0.102
GOBP_EPITHELIAL_CELL_DIFFERENTIATION_INVOLVED_IN_KIDNEY_DEVELOPMENT	0.462	1.651	0.013	0.102
GOBP_ACROSOME_ASSEMBLY	0.567	1.649	0.007	0.102
GOBP_REGULATION_OF_OSSIFICATION	0.371	1.649	0.000	0.103
GOBP_CELLULAR_RESPONSE_TO_INSULIN_STIMULUS	0.337	1.645	0.001	0.105
GOBP_PROTEIN_LOCALIZATION_TO_CYTOSKELETON	0.402	1.641	0.008	0.106
GOBP_HISTONE_H4_ACETYLATION	0.387	1.643	0.005	0.106
GOBP_REGULATION_OF_GLUCOSE_TRANSMEMBRANE_TRANSPORT	0.407	1.642	0.008	0.106
GOBP_POSITIVE_REGULATION_OF_DNA_TEMPLATED_TRANSCRIPTION_ELONGATION	0.400	1.642	0.003	0.106
GOBP_SPINDLE_LOCALIZATION	0.417	1.640	0.002	0.106

GOBP_MITOTIC_SISTER_CHROMATID_COHESION	0.481	1.641	0.020	0.106
GOBP_NUCLEAR_TRANSCRIBED_MRNA_CATABOLIC_PROCESS_NONSENSE_MEDIATED_DECAY	0.443	1.643	0.009	0.106
GOBP_T_CELL_DIFFERENTIATION_IN_THYMUS	0.384	1.643	0.003	0.106
GOBP_T_CELL_CHEMOTAXIS	0.501	1.639	0.017	0.106
GOBP_ESTABLISHMENT_OF_MITOTIC_SPINDLE_LOCALIZATION	0.457	1.639	0.010	0.107
GOBP_PROTEIN_LOCALIZATION_TO_GOLGI_APPARATUS	0.466	1.637	0.015	0.107
GOBP_PROTEIN_POLYUBIQUITINATION	0.322	1.637	0.000	0.107
GOBP_NEGATIVE_REGULATION_OF_TRANSLATIONAL_INITIATION	0.537	1.637	0.016	0.107
GOBP_CEREBRAL_CORTEX_DEVELOPMENT	0.369	1.634	0.002	0.109
GOBP_SISTER_CHROMATID_SEGREGATION	0.323	1.633	0.000	0.109
GOBP_REGULATION_OF_MICROTUBULE_BASED_PROCESS	0.330	1.633	0.000	0.109
GOBP_NEUROINFLAMMATORY_RESPONSE	0.406	1.632	0.008	0.110
GOBP_POSITIVE_REGULATION_OF_CHROMOSOME_ORGANIZATION	0.360	1.631	0.004	0.110
GOBP_MICROTUBULE_DEPOLYMERIZATION	0.440	1.631	0.019	0.110
GOBP_TYROSINE_PHOSPHORYLATION_OF_STAT_PROTEIN	0.397	1.630	0.008	0.110
GOBP_SMOOTH_MUSCLE_CELL_PROLIFERATION	0.352	1.627	0.000	0.113
GOBP_REGULATION_OF_CHROMOSOME_ORGANIZATION	0.316	1.623	0.000	0.116
GOBP_NEGATIVE_REGULATION_OF_MIRNA_METABOLIC_PROCESS	0.532	1.619	0.024	0.117
GOBP_PROTEIN_METHYLATION	0.329	1.620	0.000	0.117
GOBP_EPIGENETIC_REGULATION_OF_GENE_EXPRESSION	0.344	1.620	0.000	0.117
GOBP_PROTEIN_LOCALIZATION_TO_NUCLEUS	0.312	1.619	0.000	0.117
GOBP_POSITIVE_REGULATION_OF_CATABOLIC_PROCESS	0.298	1.620	0.000	0.118
GOBP_RAB_PROTEIN_SIGNAL_TRANSDUCTION	0.502	1.621	0.015	0.118
GOBP_RESPONSE_TO_COLD	0.436	1.616	0.025	0.119
GOBP_REGULATION_OF_HUMORAL_IMMUNE_RESPONSE_MEDIATED_BY_CIRCULATING_IMMUNOGLOBULIN	0.548	1.615	0.023	0.120
GOBP_STRIATED_MUSCLE_CELL_PROLIFERATION	0.404	1.613	0.006	0.122
GOBP_POSITIVE_REGULATION_OF_FOCAL_ADHESION_ASSEMBLY	0.518	1.611	0.024	0.123
GOBP_REGULATION_OF_MITOTIC_CELL_CYCLE_PHASE_TRANSITION	0.306	1.610	0.001	0.124
GOBP_POSITIVE_REGULATION_OF_TRANSCRIPTION_FROM_RNA_POLYMERASE_II_PROMOTER_INVOLVED_IN_CELLULAR_RESPONSE_TO_CHEMICAL_STIMULUS	0.522	1.602	0.034	0.128
GOBP_POSITIVE_REGULATION_OF_HISTONE_H3_K4_METHYLATION	0.479	1.604	0.021	0.128
GOBP_NEGATIVE_REGULATION_OF_DNA_METABOLIC_PROCESS	0.346	1.605	0.003	0.128
GOBP_MEIOTIC_CELL_CYCLE	0.320	1.601	0.001	0.128
GOBP_HISTONE_MRNA_METABOLIC_PROCESS	0.481	1.602	0.014	0.128
GOBP_CELLULAR_RESPONSE_TO_CAMP	0.410	1.603	0.009	0.128
GOBP_CENTRIOLE_CENTRIOLE_COHESION	0.557	1.604	0.024	0.128
GOBP_REGULATION_OF_NUCLEAR_TRANSCRIBED_MRNA_POLY_A_TAIL_SHORTENING	0.550	1.605	0.018	0.128
GOBP_REGULATION_OF_TELOMERE_MAINTENANCE_VIA_TELOMERE_LENGTHENING	0.394	1.602	0.014	0.129
GOBP_POSITIVE_REGULATION_OF_ENDOTHELIAL_CELL_APOPTOTIC_PROCESS	0.523	1.600	0.012	0.129
GOBP_CEREBRAL_CORTEX_RADIALLY_ORIENTED_CELL_MIGRATION	0.472	1.602	0.027	0.129
GOBP_PROTEIN_K48_LINKED_UBIQUITINATION	0.381	1.597	0.005	0.129
GOBP_DENDRITIC_SPINE_MAINTENANCE	0.533	1.598	0.022	0.129
GOBP_REGULATION_OF_TELOMERE_MAINTENANCE	0.356	1.599	0.006	0.129
GOBP_LENS_DEVELOPMENT_IN_CAMERA_TYPE_EYE	0.396	1.597	0.008	0.129
GOBP_REGULATION_OF_MITOTIC_CELL_CYCLE	0.294	1.598	0.000	0.130
GOBP_PROTEIN_DEPOLYMERIZATION	0.358	1.598	0.003	0.130

GOBP_REGULATION_OF_MUSCLE_HYPERTROPHY	0.414	1.595	0.015	0.131
GOBP_SECONDARY_PALATE_DEVELOPMENT	0.494	1.595	0.029	0.131
GOBP_AXIS_SPECIFICATION	0.391	1.594	0.009	0.131
GOBP_AUTOPHAGOSOME_ORGANIZATION	0.345	1.593	0.006	0.131
GOBP_REGULATION_OF_TRANSLATION_IN_RESPONSE_TO_STRESS	0.502	1.591	0.024	0.133
GOBP_REGULATION_OF_PROTEIN_SERINE_THREONINE_KINASE_ACTIVITY	0.305	1.591	0.000	0.133
GOBP_CEREBRAL_CORTEX_CELL_MIGRATION	0.450	1.589	0.017	0.133
GOBP_CELLULAR_RESPONSE_TO_ORGANIC_CYCLIC_COMPOUND	0.294	1.590	0.000	0.133
GOBP_ENDOTHELIAL_CELL_APOPTOTIC_PROCESS	0.414	1.589	0.013	0.133
GOBP_POSITIVE_REGULATION_OF_TRANSCRIPTION_ELONGATION_BY_RNA_POLYMERASE_II	0.402	1.587	0.011	0.135
GOBP_POSITIVE_REGULATION_OF_EPITHELIAL_TO_MESENCHYMAL_TRANSITION	0.415	1.584	0.013	0.136
GOBP_REGULATION_OF_DNA_METHYLATION_DEPENDENT_HETEROCHROMATIN_FORMATION	0.534	1.585	0.028	0.136
GOBP_NEGATIVE_REGULATION_OF_TRANSMEMBRANE_RECEPTOR_PROTEIN_SERINE_THREONINE_KINASE_SIGNALING_PATHWAY	0.348	1.585	0.002	0.137
GOBP_NEGATIVE_REGULATION_OF_TRANSFORMING_GROWTH_FACTOR_BETA_RECEPTOR_SIGNALING_PATHWAY	0.367	1.584	0.008	0.137
GOBP_GLUCOSE_IMPORT	0.399	1.583	0.010	0.138
GOBP_CELLULAR_RESPONSE_TO_STARVATION	0.330	1.582	0.000	0.138
GOBP_POSITIVE_REGULATION_OF_GTPASE_ACTIVITY	0.311	1.577	0.000	0.139
GOBP_NEGATIVE_REGULATION_OF_PROTEIN_CONTAINING_COMPLEX_DISASSEMBLY	0.377	1.574	0.010	0.139
GOBP_INTRACELLULAR_RECEPTOR_SIGNALING_PATHWAY	0.313	1.577	0.000	0.139
GOBP_RESPONSE_TO_PROTOZOAN	0.480	1.572	0.026	0.140
GOBP_REGULATION_OF_B_CELL_MEDIATED_IMMUNITY	0.382	1.575	0.017	0.140
GOBP_NUCLEOBASE_CONTAINING_COMPOUND_TRANSPORT	0.317	1.575	0.003	0.140
GOBP_NEGATIVE_REGULATION_OF_PRODUCTION_OF_MOLECULAR_MEDIATOR_OF_IMMUNE_RESPONSE	0.427	1.573	0.029	0.140
GOBP_SOMATIC_STEM_CELL_POPULATION_MAINTENANCE	0.398	1.576	0.018	0.140
GOBP_NEGATIVE_REGULATION_OF_UBIQUITIN_DEPENDENT_PROTEIN_CATABOLIC_PROCESS	0.406	1.578	0.012	0.140
GOBP_PLATELET_DERIVED_GROWTH_FACTOR_RECEPTOR_SIGNALING_PATHWAY	0.406	1.573	0.018	0.140
GOBP_REGULATION_OF_SISTER_CHROMATID_COHESION	0.485	1.573	0.038	0.140
GOBP_P38MAPK_CASCADE	0.409	1.576	0.009	0.140
GOBP_CELLULAR_RESPONSE_TO_PEPTIDE	0.303	1.575	0.000	0.140
GOBP_REGULATION_OF_MIRNA_METABOLIC_PROCESS	0.375	1.572	0.012	0.140
GOBP_ACTIVATION_OF_INNATE_IMMUNE_RESPONSE	0.393	1.580	0.010	0.140
GOBP_POSITIVE_REGULATION_OF_GLUCOSE_METABOLIC_PROCESS	0.440	1.579	0.027	0.140
GOBP_OSSIFICATION	0.302	1.575	0.000	0.140
GOBP_REGULATION_OF_DNA_TEMPLATED_TRANSCRIPTION_ELONGATION	0.346	1.578	0.002	0.140
GOBP_REGULATION_OF_LYMPHOCYTE_DIFFERENTIATION	0.318	1.572	0.001	0.140
GOBP_MRNA_CIS_SPLICING_VIA_SPLICEOSOME	0.489	1.578	0.025	0.140
GOBP_HIPPOCAMPUS_DEVELOPMENT	0.390	1.571	0.011	0.140
GOBP_MUSCLE_CELL_APOPTOTIC_PROCESS	0.393	1.579	0.015	0.140
GOBP_B_CELL_ACTIVATION	0.310	1.578	0.000	0.140
GOBP_EPITHELIAL_CELL_APOPTOTIC_PROCESS	0.346	1.570	0.003	0.140
GOBP_REGULATION_OF_PROTEIN_ACETYLATION	0.366	1.570	0.006	0.141
GOBP_NEGATIVE_REGULATION_OF_BIOMINERALIZATION	0.506	1.569	0.033	0.141
GOBP_EPITHELIAL_CELL_DEVELOPMENT	0.320	1.568	0.003	0.142
GOBP_CYTOKINESIS	0.325	1.566	0.000	0.143

GOBP_CENTRAL_NERVOUS_SYSTEM_NEURON_AXONOGENESIS	0.477	1.565	0.026	0.143
GOBP_POSITIVE_REGULATION_OF_MACROAUTOPHAGY	0.368	1.565	0.006	0.143
GOBP_GLOMERULAR_EPITHELIUM_DEVELOPMENT	0.511	1.566	0.036	0.143
GOBP_SALIVARY_GLAND_DEVELOPMENT	0.478	1.566	0.027	0.143
GOBP_ACTOMYOSIN_STRUCTURE_ORGANIZATION	0.327	1.561	0.003	0.144
GOBP_MEIOSIS_II_CELL_CYCLE_PROCESS	0.535	1.562	0.033	0.144
GOBP_NITRIC_OXIDE_SYNTHASE_BIOSYNTHETIC_PROCESS	0.498	1.563	0.030	0.144
GOBP_NEGATIVE_REGULATION_OF_OSTEOCLAST_DIFFERENTIATION	0.457	1.560	0.024	0.144
GOBP_REGULATION_OF_HISTONE_H3_K9_METHYLATION	0.466	1.562	0.025	0.144
GOBP_STRIATED_MUSCLE_TISSUE_DEVELOPMENT	0.322	1.563	0.003	0.144
GOBP_REGULATION_OF_CELL_CYCLE_G1_S_PHASE_TRANSITION	0.315	1.562	0.000	0.145
GOBP_NEGATIVE_REGULATION_OF_CELL_MATRIX_ADHESION	0.452	1.560	0.026	0.145
GOBP_REGULATION_OF_SMALL_GTPASE_MEDIATED_SIGNAL_TRANSDUCTION	0.303	1.560	0.000	0.145
GOBP_RESPONSE_TO_INSULIN	0.309	1.559	0.000	0.145
GOBP_POSITIVE_REGULATION_OF_BLOOD_VESSEL_ENDOTHELIAL_CELL_MIGRATION	0.408	1.557	0.018	0.147
GOBP_STRESS_GRANULE_ASSEMBLY	0.472	1.553	0.024	0.147
GOBP_POSITIVE_REGULATION_OF_SMOOTH_MUSCLE_CELL_PROLIFERATION	0.365	1.552	0.014	0.147
GOBP_CHROMOSOME_SEGREGATION	0.293	1.556	0.000	0.147
GOBP_CENTROMERE_COMPLEX_ASSEMBLY	0.435	1.553	0.035	0.147
GOBP_SMOOTH_MUSCLE_CELL_APOPTOTIC_PROCESS	0.495	1.552	0.030	0.148
GOBP_NEGATIVE_REGULATION_OF_HEMOPOIESIS	0.350	1.556	0.009	0.148
GOBP_REGULATION_OF_PLATELET_DERIVED_GROWTH_FACTOR_RECEPTOR_SIGNALI NG_PATHWAY	0.503	1.556	0.037	0.148
GOBP_NON_MEMBRANE_BOUNDED_ORGANELLE_ASSEMBLY	0.291	1.553	0.000	0.148
GOBP_POSITIVE_REGULATION_OF_CYTOSKELETON_ORGANIZATION	0.323	1.553	0.001	0.148
GOBP_REGULATION_OF_KERATINOCYTE_PROLIFERATION	0.447	1.554	0.019	0.148
GOBP_NEGATIVE_REGULATION_OF_T_CELL_PROLIFERATION	0.380	1.553	0.015	0.148
GOBP_NUCLEAR_CHROMOSOME_SEGREGATION	0.298	1.554	0.000	0.148
GOBP_PROTEIN_LOCALIZATION_TO_MICROTUBULE_ORGANIZING_CENTER	0.435	1.553	0.031	0.149
GOBP_CENTRAL_NERVOUS_SYSTEM_PROJECTION_NEURON_AXONOGENESIS	0.516	1.548	0.039	0.149
GOBP_REGULATION_OF_INTRACELLULAR_TRANSPORT	0.298	1.550	0.000	0.149
GOBP_CELLULAR_RESPONSE_TO_RADIATION	0.316	1.548	0.000	0.149
GOBP_ATTACHMENT_OF_SPINDLE_MICROTUBULES_TO_KINETOCHORE	0.402	1.548	0.016	0.149
GOBP_FOREBRAIN_CELL_MIGRATION	0.410	1.549	0.016	0.149
GOBP_POSITIVE_REGULATION_OF_PROTEIN_EXPORT_FROM_NUCLEUS	0.505	1.547	0.034	0.149
GOBP_CELLULAR_RESPONSE_TO_HEAT	0.381	1.549	0.016	0.150
GOBP_REGULATION_OF_EPITHELIAL_CELL_APOPTOTIC_PROCESS	0.363	1.549	0.006	0.150
GOBP_INORGANIC_ION_IMPORT_ACROSS_PLASMA_MEMBRANE	0.369	1.546	0.011	0.150
GOBP_CELL_PART_MORPHOGENESIS	0.281	1.546	0.000	0.150
GOBP_POSITIVE_REGULATION_OF_REACTIVE_OXYGEN_SPECIES_BIOSYNTHETIC_PRO CESS	0.518	1.546	0.037	0.150
GOBP_NEGATIVE_REGULATION_OF_CELLULAR_CATABOLIC_PROCESS	0.312	1.546	0.000	0.150
GOBP_POSITIVE_REGULATION_OF_STEM_CELL_POPULATION_MAINTENANCE	0.400	1.543	0.025	0.152
GOBP_FAT_CELL_DIFFERENTIATION	0.313	1.541	0.000	0.153
GOBP_REGULATION_OF_HISTONE_H3_K4_METHYLATION	0.414	1.541	0.023	0.153
GOBP_NEGATIVE_REGULATION_OF_CELL_CYCLE_G1_S_PHASE_TRANSITION	0.363	1.540	0.012	0.153
GOBP_POST_EMBRYONIC_DEVELOPMENT	0.358	1.539	0.002	0.153
GOBP_REGULATION_OF_CHROMOSOME_SEGREGATION	0.329	1.541	0.003	0.153

GOBP_FOCAL_ADHESION_ASSEMBLY	0.366	1.540	0.010	0.153
GOBP_HIPPO_SIGNALING	0.423	1.540	0.024	0.153
GOBP_ASTROCYTE_DEVELOPMENT	0.433	1.541	0.018	0.153
GOBP_POSITIVE_REGULATION_OF_CELLULAR_RESPONSE_TO_TRANSFORMING_GROWTH_FACTOR_BETA_STIMULUS	0.450	1.538	0.034	0.153
GOBP_REGULATION_OF_GLUCOSE_IMPORT	0.410	1.537	0.016	0.153
GOBP_MICROTUBULE_ANCHORING	0.451	1.542	0.037	0.153
GOBP_REGULATION_OF_INTRACELLULAR_PROTEIN_TRANSPORT	0.305	1.537	0.000	0.154
GOBP_REGULATION_OF_MUSCLE_ADAPTATION	0.378	1.538	0.021	0.154
GOBP_EPIBOLY	0.436	1.538	0.022	0.154
GOBP_POSITIVE_REGULATION_OF_CARBOHYDRATE_METABOLIC_PROCESS	0.382	1.535	0.017	0.154
GOBP_NEGATIVE_REGULATION_OF_KERATINOCYTE_PROLIFERATION	0.504	1.535	0.031	0.154
GOBP_CHONDROCYTE_PROLIFERATION	0.528	1.534	0.041	0.155
GOBP_POSITIVE_REGULATION_OF_ORGANELLE_ORGANIZATION	0.285	1.535	0.001	0.155
GOBP_NEGATIVE_REGULATION_OF_MYELOID_LEUKOCYTE_DIFFERENTIATION	0.396	1.534	0.016	0.155
GOBP_POSITIVE_REGULATION_OF_MUSCLE_HYPERTROPHY	0.478	1.535	0.037	0.155
GOBP_ENDOCRINE_HORMONE_SECRETION	0.426	1.531	0.033	0.157
GOBP_REGULATION_OF_PROTEIN_DEPOLYMERIZATION	0.370	1.531	0.020	0.158
GOBP_NEGATIVE_REGULATION_OF_IMMUNE_EFFECTOR_PROCESS	0.341	1.528	0.008	0.160
GOBP_NEGATIVE_REGULATION_OF_EPITHELIAL_CELL_DIFFERENTIATION	0.442	1.527	0.030	0.161
GOBP_NEGATIVE_REGULATION_OF_PROTEIN_SERINE_THREONINE_KINASE_ACTIVITY	0.340	1.527	0.010	0.161
GOBP_REGULATION_OF_CELL_DIVISION	0.320	1.527	0.006	0.161
GOBP_SPINDLE_ASSEMBLY	0.321	1.525	0.000	0.161
GOBP_REGULATION_OF_CIRCADIAN_RHYTHM	0.343	1.526	0.012	0.162
GOBP_REGULATION_OF_ACTOMYOSIN_STRUCTURE_ORGANIZATION	0.355	1.525	0.017	0.162
GOBP_NEGATIVE_REGULATION_OF_ADAPTIVE_IMMUNE_RESPONSE	0.389	1.523	0.018	0.163
GOBP_POSITIVE_REGULATION_OF_CELLULAR_CARBOHYDRATE_METABOLIC_PROCESS	0.415	1.524	0.018	0.163
GOBP_REGULATION_OF_NEUROBLAST_PROLIFERATION	0.454	1.521	0.037	0.164
GOBP_POSITIVE_REGULATION_OF_SUPRAMOLECULAR_FIBER_ORGANIZATION	0.318	1.521	0.007	0.164
GOBP_CELL_SUBSTRATE_JUNCTION_ORGANIZATION	0.347	1.522	0.004	0.165
GOBP_POSITIVE_REGULATION_OF_PROTEIN_KINASE_ACTIVITY	0.289	1.520	0.000	0.165
GOBP_POSITIVE_REGULATION_OF_LYMPHOCYTE_DIFFERENTIATION	0.329	1.519	0.006	0.166
GOBP_POSITIVE_REGULATION_OF_DNA_METABOLIC_PROCESS	0.292	1.518	0.001	0.167
GOBP_POSITIVE_REGULATION_OF_MITOTIC_CELL_CYCLE	0.332	1.518	0.008	0.167
GOBP_BLOOD_VESSEL_MORPHOGENESIS	0.277	1.517	0.001	0.167
GOBP_POSITIVE_REGULATION_OF_TYROSINE_PHOSPHORYLATION_OF_STAT_PROTEIN	0.395	1.517	0.032	0.168
GOBP_CELL_CELL_JUNCTION_ASSEMBLY	0.341	1.516	0.015	0.168
GOBP_MITOTIC_NUCLEAR_DIVISION	0.287	1.512	0.000	0.171
GOBP_FC_EPSILON_RECEPTOR_SIGNALING_PATHWAY	0.459	1.512	0.039	0.171
GOBP_RNA_3_END_PROCESSING	0.340	1.512	0.013	0.172
GOBP_B_CELL_DIFFERENTIATION	0.322	1.512	0.006	0.172
GOBP_EPITHELIAL_TO_MESENCHYMAL_TRANSITION	0.327	1.510	0.002	0.172
GOBP_CELLULAR_RESPONSE_TO_EXTERNAL_STIMULUS	0.296	1.511	0.004	0.172
GOBP_REGULATION_OF_PROTEIN_POLYUBIQUITINATION	0.443	1.510	0.039	0.173
GOBP_POSITIVE_REGULATION_OF_RNA_SPLICING	0.417	1.510	0.023	0.173
GOBP_IRON_ION_TRANSPORT	0.390	1.508	0.026	0.173
GOBP_PROTEASOMAL_PROTEIN_CATABOLIC_PROCESS	0.277	1.505	0.000	0.174

GOBP_KINETOCHORE_ORGANIZATION	0.465	1.505	0.030	0.174
GOBP_LIMBIC_SYSTEM_DEVELOPMENT	0.361	1.506	0.014	0.174
GOBP_MEIOTIC_CELL_CYCLE_PROCESS	0.311	1.506	0.007	0.174
GOBP_MIRNA_MEDIATED_GENE_SILENCING_BY_INHIBITION_OF_TRANSLATION	0.519	1.507	0.043	0.175
GOBP_POSITIVE_REGULATION_OF_CHROMOSOME_SEGREGATION	0.453	1.506	0.048	0.175
GOBP_REGULATION_OF_MICROTUBULE_DEPOLYMERIZATION	0.450	1.504	0.048	0.175
GOBP_MUSCLE_ADAPTATION	0.349	1.506	0.008	0.175
GOBP_ESTABLISHMENT_OF_PROTEIN_LOCALIZATION_TO_PLASMA_MEMBRANE	0.374	1.502	0.016	0.177
GOBP_POSITIVE_REGULATION_OF_MUSCLE_CELL_APOPTOTIC_PROCESS	0.465	1.502	0.032	0.177
GOBP_DEVELOPMENTAL_GROWTH	0.276	1.502	0.000	0.177
GOBP_MITOTIC_CELL_CYCLE_CHECKPOINT_SIGNALING	0.318	1.500	0.007	0.177
GOBP_REGULATION_OF_NEURON_MIGRATION	0.445	1.501	0.037	0.177
GOBP_LENS_FIBER_CELL_DIFFERENTIATION	0.450	1.500	0.046	0.177
GOBP_CELLULAR_RESPONSE_TO_HORMONE_STIMULUS	0.276	1.499	0.001	0.178
GOBP_REGULATION_OF_HEMATOPOIETIC_PROGENITOR_CELL_DIFFERENTIATION	0.408	1.498	0.025	0.178
GOBP_REGULATION_OF_CELLULAR_MACROMOLECULE_BIOSYNTHETIC_PROCESS	0.277	1.499	0.000	0.178
GOBP_TELENCEPHALON_DEVELOPMENT	0.305	1.497	0.004	0.179
GOBP_SOMATIC_DIVERSIFICATION_OF_IMMUNE_RECEPTORS	0.346	1.497	0.024	0.179
GOBP_POSITIVE_REGULATION_OF_PROTEIN_SERINE_THREONINE_KINASE_ACTIVITY	0.304	1.495	0.001	0.179
GOBP_INTERLEUKIN_6_MEDIATED_SIGNALING_PATHWAY	0.494	1.494	0.049	0.179
GOBP_POSITIVE_REGULATION_OF_KINASE_ACTIVITY	0.280	1.493	0.001	0.180
GOBP_GLIAL_CELL_ACTIVATION	0.388	1.493	0.032	0.180
GOBP_CARBOHYDRATE_TRANSMEMBRANE_TRANSPORT	0.336	1.495	0.020	0.180
GOBP_NEGATIVE_REGULATION_OF_PROTEASOMAL_UBIQUITIN_DEPENDENT_PROTEIN_CATABOLIC_PROCESS	0.419	1.493	0.036	0.180
GOBP_FC_RECEPTOR_SIGNALING_PATHWAY	0.372	1.493	0.030	0.180
GOBP_REGULATION_OF_LYMPHOCYTE_ACTIVATION	0.279	1.494	0.001	0.180
GOBP_APICAL_JUNCTION_ASSEMBLY	0.386	1.495	0.025	0.180
GOBP_KILLING_OF_CELLS_OF_ANOTHER_ORGANISM	0.444	1.495	0.035	0.180
GOBP_REGULATION_OF_TELOMERE_CAPPING	0.448	1.495	0.038	0.180
GOBP_ESTABLISHMENT_OF_CELL_POLARITY	0.318	1.492	0.004	0.180
GOBP_POSITIVE_REGULATION_OF_MITOTIC_CELL_CYCLE_PHASE_TRANSITION	0.346	1.491	0.015	0.181
GOBP_JNK_CASCADE	0.316	1.490	0.012	0.183
GOBP_NEGATIVE_REGULATION_OF_LYMPHOCYTE_MEDIATED_IMMUNITY	0.379	1.489	0.023	0.183
GOBP_MICROTUBULE_CYTOSKELETON_ORGANIZATION_INVOLVED_IN_MITOSIS	0.308	1.484	0.006	0.184
GOBP_CHROMOSOME_ORGANIZATION_INVOLVED_IN_MEIOTIC_CELL_CYCLE	0.358	1.487	0.031	0.184
GOBP_BIOLOGICAL_PROCESS_INVOLVED_IN_INTERACTION_WITH_SYMBIONT	0.330	1.484	0.012	0.184
GOBP_OOCYTE_MATURATION	0.432	1.487	0.043	0.184
GOBP_CELLULAR_RESPONSE_TO_EXTRACELLULAR_STIMULUS	0.297	1.485	0.004	0.184
GOBP_IN_UTERO_EMBRYONIC_DEVELOPMENT	0.281	1.484	0.000	0.184
GOBP_MODULATION_BY_HOST_OF_SYMBIONT_PROCESS	0.353	1.486	0.018	0.185
GOBP_MUSCLE_TISSUE_DEVELOPMENT	0.288	1.485	0.003	0.185
GOBP_PROTEIN_TARGETING_TO_LYSOSOME	0.441	1.485	0.041	0.185
GOBP_CELLULAR_RESPONSE_TO_STEROID_HORMONE_STIMULUS	0.305	1.484	0.003	0.185
GOBP_RESPONSE_TO_UV	0.317	1.484	0.006	0.185
GOBP_TRANSFORMING_GROWTH_FACTOR_BETA_PRODUCTION	0.414	1.479	0.046	0.185
GOBP_POSITIVE_REGULATION_OF_CELLULAR_COMPONENT_BIOGENESIS	0.277	1.479	0.001	0.185
GOBP_SMALL_GTPASE_MEDIATED_SIGNAL_TRANSDUCTION	0.273	1.480	0.001	0.185

GOBP_RESPONSE_TO_STARVATION	0.305	1.479	0.009	0.186
GOBP_REGULATION_OF_HEART_GROWTH	0.382	1.480	0.029	0.186
GOBP_MICROTUBULE_POLYMERIZATION_OR_DEPOLYMERIZATION	0.327	1.480	0.009	0.186
GOBP_TELOMERE_CAPPING	0.392	1.481	0.038	0.186
GOBP_INOSITOL_PHOSPHATE_MEDIATED_SIGNALING	0.388	1.480	0.032	0.186
GOBP_REGULATION_OF_LEUKOCYTE_DIFFERENTIATION	0.288	1.482	0.003	0.186
GOBP_POSITIVE_REGULATION_OF_OSSIFICATION	0.391	1.482	0.035	0.186
GOBP_REGULATION_OF_NEUROINFLAMMATORY_RESPONSE	0.443	1.480	0.044	0.186
GOBP_REGULATION_OF_T_CELL_ACTIVATION	0.282	1.481	0.000	0.186
GOBP_AMYLOID_BETA_CLEARANCE	0.411	1.478	0.041	0.187
GOBP_REGULATION_OF_MIRNA_TRANSCRIPTION	0.368	1.476	0.033	0.188
GOBP_NEGATIVE_REGULATION_OF_INTRACELLULAR_SIGNAL_TRANSDUCTION	0.272	1.476	0.000	0.188
GOBP_TRANSMEMBRANE_RECEPTOR_PROTEIN_SERINE_THREONINE_KINASE_SIGNAL ING_PATHWAY	0.286	1.476	0.001	0.188
GOBP_POST_TRANSCRIPTIONAL_REGULATION_OF_GENE_EXPRESSION	0.271	1.475	0.001	0.188
GOBP_NEGATIVE_REGULATION_OF_LEUKOCYTE_MEDIATED_IMMUNITY	0.360	1.475	0.011	0.189
GOBP_STRESS_ACTIVATED_PROTEIN_KINASE_SIGNALING_CASCADE	0.295	1.473	0.006	0.189
GOBP_NEGATIVE_REGULATION_OF_TELOMERE_MAINTENANCE	0.401	1.473	0.036	0.189
GOBP_CELLULAR_RESPONSE_TO_IONIZING_RADIATION	0.354	1.472	0.022	0.191
GOBP_GOLGI_TO_PLASMA_MEMBRANE_PROTEIN_TRANSPORT	0.392	1.471	0.038	0.191
GOBP_CELLULAR_RESPONSE_TO_ALCOHOL	0.350	1.471	0.022	0.191
GOBP_REGULATION_OF_GTPASE_ACTIVITY	0.280	1.470	0.005	0.192
GOBP_MONONUCLEAR_CELL_DIFFERENTIATION	0.273	1.470	0.000	0.192
GOBP_CELLULAR_RESPONSE_TO_LIGHT_STIMULUS	0.327	1.469	0.012	0.192
GOBP_REGULATION_OF_OSTEOCLAST_DIFFERENTIATION	0.365	1.469	0.028	0.193
GOBP_CELL_MIGRATION_INVOLVED_IN_SPROUTING_ANGIOGENESIS	0.395	1.468	0.037	0.193
GOBP_STEROID_HORMONE_MEDIATED_SIGNALING_PATHWAY	0.318	1.467	0.018	0.194
GOBP_TIGHT_JUNCTION_ORGANIZATION	0.378	1.466	0.034	0.195
GOBP_POSITIVE_REGULATION_OF_SIGNALING_RECEPTOR_ACTIVITY	0.400	1.465	0.048	0.196
GOBP_REGULATION_OF_NEURONAL_SYNAPTIC_PLASTICITY	0.399	1.463	0.038	0.197
GOBP_REGULATION_OF_TRANSLATIONAL_INITIATION	0.339	1.463	0.012	0.198
GOBP_CELLULAR_RESPONSE_TO_GLUCOSE_STARVATION	0.370	1.457	0.029	0.198
GOBP_NEGATIVE_REGULATION_OF_MICROTUBULE_POLYMERIZATION_OR_DEPOLYM ERIZATION	0.415	1.458	0.050	0.198
GOBP_REGULATION_OF_MICROTUBULE_CYTOSKELETON_ORGANIZATION	0.313	1.459	0.014	0.198
GOBP_POSITIVE_REGULATION_OF_BONE_MINERALIZATION	0.408	1.457	0.046	0.198
GOBP_AXONAL_TRANSPORT	0.357	1.458	0.044	0.198
GOBP_REGULATION_OF_T_HELPER_CELL_DIFFERENTIATION	0.398	1.458	0.042	0.198
GOBP_AXO_DENDRITIC_TRANSPORT	0.348	1.462	0.032	0.198
GOBP_NEGATIVE_REGULATION_OF_PROTEASOMAL_PROTEIN_CATABOLIC_PROCESS	0.376	1.458	0.031	0.198
GOBP_NEGATIVE_REGULATION_OF_NEURON_DIFFERENTIATION	0.378	1.461	0.040	0.199
GOBP_REGULATION_OF_EPITHELIAL_CELL_DIFFERENTIATION	0.325	1.460	0.016	0.199
GOBP_NEGATIVE_REGULATION_OF_CYTOSKELETON_ORGANIZATION	0.315	1.461	0.018	0.199
GOBP_REGULATION_OF_BLOOD_VESSEL_ENDOTHELIAL_CELL_MIGRATION	0.343	1.459	0.027	0.199
GOBP_CARTILAGE_DEVELOPMENT	0.308	1.459	0.014	0.199
GOBP_CYTOSOLIC_TRANSPORT	0.301	1.456	0.014	0.199
GOBP_POSITIVE_REGULATION_OF_HEMOPOIESIS	0.300	1.456	0.010	0.199
GOBP_SOMATIC_DIVERSIFICATION_OF_IMMUNOGLOBULINS_INVOLVED_IN_IMMUN E_RESPONSE	0.372	1.456	0.037	0.199

GOBP_CELLULAR_RESPONSE_TO_TOPOLOGICALLY_INCORRECT_PROTEIN	0.321	1.461	0.013	0.199
GOBP_DOUBLE_STRAND_BREAK_REPAIR	0.281	1.460	0.007	0.199
GOBP_POST_GOLGI_VESICLE_MEDIATED_TRANSPORT	0.328	1.460	0.009	0.199
GOBP_ORGANELLE_FISSION	0.268	1.455	0.000	0.199
